# Phenotyping the hidden half: combining UAV phenotyping and machine learning to predict barley root traits in the field

**DOI:** 10.1093/jxb/eraf268

**Published:** 2025-06-28

**Authors:** Samir Alahmad, Daniel Smith, Christina Katsikis, Zachary Aldiss, Stephanie M Brunner, Sarah V Meer, Lotus Meijer, Bita Heidariask, Karine Chenu, Scott Chapman, Andries B Potgieter, Anton Wasson, Silvina Baraibar, Jayfred Godoy, David Moody, Hannah Robinson, Lee T Hickey

**Affiliations:** Queensland Alliance for Agriculture and Food Innovation (QAAFI), The University of Queensland (UQ), Brisbane, QLD 4072, Australia; School of Agriculture and Food Sustainability, UQ, St Lucia, QLD 4072, Australia; Queensland Alliance for Agriculture and Food Innovation (QAAFI), The University of Queensland (UQ), Brisbane, QLD 4072, Australia; Queensland Alliance for Agriculture and Food Innovation (QAAFI), The University of Queensland (UQ), Brisbane, QLD 4072, Australia; Queensland Alliance for Agriculture and Food Innovation (QAAFI), The University of Queensland (UQ), Brisbane, QLD 4072, Australia; Queensland Alliance for Agriculture and Food Innovation (QAAFI), The University of Queensland (UQ), Brisbane, QLD 4072, Australia; Queensland Alliance for Agriculture and Food Innovation (QAAFI), The University of Queensland (UQ), Brisbane, QLD 4072, Australia; Centre for Crop Science, UQ, QAAFI, Gatton, QLD, Australia; Centre for Crop Science, UQ, QAAFI, Gatton, QLD, Australia; Queensland Alliance for Agriculture and Food Innovation (QAAFI), The University of Queensland (UQ), Brisbane, QLD 4072, Australia; School of Agriculture and Food Sustainability, UQ, St Lucia, QLD 4072, Australia; Centre for Crop Science, UQ, QAAFI, Gatton, QLD, Australia; Agriculture & Food, Commonwealth Scientific and Industrial Research Organisation, Canberra, ACT 2601, Australia; InterGrain Pty. Ltd, Perth, WA 6163, Australia; InterGrain Pty. Ltd, Perth, WA 6163, Australia; InterGrain Pty. Ltd, Perth, WA 6163, Australia; Queensland Alliance for Agriculture and Food Innovation (QAAFI), The University of Queensland (UQ), Brisbane, QLD 4072, Australia; Department of Plant Breeding, Hochschule Geisenheim University, D-65366 Geisenheim, Germany; Queensland Alliance for Agriculture and Food Innovation (QAAFI), The University of Queensland (UQ), Brisbane, QLD 4072, Australia; University of Illinois Urbana-Champaign, USA

**Keywords:** Crop improvement, haplotype mapping, local genomic estimated breeding values, machine learning, partial least squares regression, prediction, random forest, root phenotyping, UAV phenotyping, vegetation indices

## Abstract

Improving crop root systems for enhanced adaptation and productivity remains challenging due to limitations in scalable non-destructive phenotyping approaches, inconsistent translation of root phenotypes from controlled environments to the field, and a lack of understanding of genetic controls. This study serves as a proof of concept, evaluating a panel of Australian barley breeding lines and cultivars in field experiments conducted across two contrasting environments. A diverse subset of 20 genotypes was subjected to ground-based root and shoot phenotyping at key growth stages, and this dataset was used in combination with unmanned aerial vehicle (UAV)-captured vegetation indices (VIs) to train machine learning models to predict root distribution and above-ground biomass for the untested panel comprising 544 genotypes across the two seasons. Unlike previous root studies that have focused on above-ground traits or indirect proxies, this approach predicts root traits in the field using machine learning. Haplotype-based mapping using predicted root and shoot traits in the broader panel revealed key genomic regions. These include novel regions, previously reported root quantitative trait loci, and *EGT2—*a recently cloned gene that regulates root gravitropism in barley. This scalable phenotyping approach offers opportunities to advance root research across crops and support the development of future varieties adapted to changing climates.

## Teaser

Integrating drone-based phenotyping and machine learning enables non-destructive prediction of root system traits, offering a novel approach to support root research and improve crop resilience under climate change.

## Introduction

Optimization of root system architecture (RSA) has been flagged as a key breeding target to develop climate-resilient crops for the future (Ober *et al.*, 2021). RSA refers to the spatial and temporal distribution of roots, which influences the uptake of water and nutrient resources in the soil to sustain and maximize crop performance (Koevoets *et al.*, 2016). However, the value of different root architectures is highly context dependent and driven by the environmental and management conditions experienced by the crop (Voss-Fels *et al.*, 2018; Shazadi *et al.*, 2024). For example, root proliferation in superficial soil layers could be advantageous in environments that experience sporadic rainfall and also facilitate access to nutrients, including phosphorus (Henry *et al.*, 2010; Lynch, 2019; van der Bom *et al.*, 2023). On the other hand, deeper root systems have the potential to enhance access to stored moisture to improve yield under terminal drought conditions (Rich *et al.*, 2016; Alahmad *et al.*, 2019). For instance, modelling studies suggest that wheat yield could increase by 55 kg ha^–1^ for each additional millimetre of water extracted during grain filling (Manschadi *et al.*, 2006; Christopher *et al.*, 2013). Customizing crop root systems for specific agroecological contexts has the potential to achieve enhanced water and nutrient efficiency, sustainability, and profitability (Ober *et al.*, 2021).

Historically, RSA traits were overlooked in crop improvement programmes, where breeders have focused on above-ground traits, including flowering time and height (Den Herder *et al.*, 2010; Borrell *et al.*, 2014; Christopher *et al.*, 2016; Robinson *et al.*, 2018; Ober *et al.*, 2021). The primary reason for this has been the difficulty in directly measuring RSA traits in the field. While innovations in field-based root phenotyping methods, including ‘mini-rhizotrons’, ‘shovelomics’, and ‘core break’ methods (Trachsel *et al.*, 2011; Wasson *et al.*, 2014; York *et al.*, 2018, Preprint), have provided advances and offer valuable insights, they are often constrained by population size and labour intensity (Trachsel *et al.*, 2011; Wasson *et al.*, 2014; York *et al.*, 2018, Preprint).

High-throughput, cost-effective techniques under controlled conditions, such as ‘paper pouch’ (Hund *et al.*, 2009), ‘clear pot’ (Richard *et al.*, 2015), and rhizobox methods (Joshi *et al.*, 2017) have been developed to overcome the root phenotyping bottleneck and have enabled genomic discoveries for seminal RSA traits in durum wheat (Cane *et al.*, 2014; Maccaferri *et al.*, 2016; Alahmad *et al.*, 2019), bread wheat (Atkinson *et al.*, 2015), and barley (Robinson *et al.*, 2016). However, root phenotypes observed in early growth stages are less representative of the phenotype of mature roots in the field and often have weak to moderate associations (Mace *et al.*, 2012; Borrell *et al.*, 2014; Maccaferri *et al.*, 2016; El Hassouni *et al.*, 2018; Alahmad *et al.*, 2020). Furthermore, while several studies have identified quantitative trait loci (QTL) associated with barley seedling RSA traits under controlled conditions (Chloupek *et al.*, 2006; Naz *et al.*, 2014; Arifuzzaman *et al.*, 2016; Robinson *et al.*, 2016; Jia *et al.*, 2019), the genetic drivers of mature RSA traits under field conditions remain poorly understood.

Unmanned aerial vehicle (UAV) or drone technologies are transforming crop phenotyping, providing high-resolution, scalable data for canopy traits (Kyratzis *et al.*, 2017). For example, UAV-captured vegetation indices (VIs) precisely estimated sorghum leaf area and canopy cover (Potgieter *et al.*, 2017; Zhao *et al.*, 2021). Further, remote sensing enabled accurate prediction of water- and nitrogen-use efficiency and the assessment of canopy temperature and stomatal conductance, in wheat and maize (Zhang *et al.*, 2019; Yang *et al.*, 2020; Brewer *et al.*, 2022). These technologies improve phenotyping by enhancing the precision of traits such as canopy coverage, enabling detection of traits beyond human perception such as canopy temperature, and allowing robust measurement of complex traits such as canopy biomass, revealing new opportunities for crop improvement. When combined with machine learning as a complementary tool, their predictive power is significantly enhanced, enabling the detection of subtle patterns and relationships which would otherwise remain hidden. Wang *et al.* (2023) described two main approaches for the prediction of above-ground biomass using remote sensing. The first integrates remote sensing data into crop simulation models, such as APSIM, to improve the accuracy of biomass prediction (Hammer *et al.*, 2010; Yang *et al.*, 2021; Sadeh *et al.*, 2024). The second focuses on machine learning models, such as partial least squares regression (PLSR) and Random Forest (RF), which leverage UAV data to predict traits accurately. PLSR is a multivariate regression method adept at handling a large number of predictor variables, even when they exhibit a high degree of co-linearity (Wold, 1975). Historically, PLSR has been widely used in remote sensing and spectral datasets with strong linear association (Robles-Zazueta *et al.*, 2021). Similarly, RF is an ensemble learning method that builds multiple decision trees to enhance prediction accuracy and effectively handle complex datasets (Breiman, 2001). Both methods have been widely applied to leverage UAV-captured data to predict above-ground biomass with high accuracy (Han *et al.*, 2019; Huntington *et al.*, 2020; Masjedi *et al.*, 2020). These indirect approaches are non-destructive and repeatable, with decreased susceptibility to human error and improved accuracies. Furthermore, due to the potential scalability, they are well suited to breeding programmes and large-scale variety selection initiatives, and provide the opportunity to explore the genetic architecture of complex traits due to increased population size (Smith *et al.*, 2021). Consequently, extensive research has investigated indirect methods to predict biomass accumulation using UAV phenotyping, including the use of multispectral (Liu *et al.*, 2019), hyperspectral (Yoosefzadeh-Najafabadi *et al.*, 2021), and thermal imagery (Pinto and Reynolds, 2015). Recent advances have also demonstrated that low-cost phenomic prediction using high-throughput tools such as near-infrared spectroscopy can capture genetic variation among parental lines and serve as a substitute for genotyping, offering scalable prediction strategies in both hybrid and inbred breeding programmes (Rincent *et al.*, 2025).

Previous studies have also explored the potential to use UAV-captured VIs or canopy temperature as ‘proxy traits’ to identify genotypes that have improved access to soil water through enhanced RSA, and report promising correlations (Lopes and Reynolds, 2010; Pinto *et al.*, 2010; Pinto and Reynolds, 2015; Li *et al.*, 2019). This relationship is potentially driven by leaf area, stomatal conductance, and root access to soil moisture (Ober *et al.*, 2021). To explore water supply and demand, earlier studies demonstrated the potential for using spectral reflectance in the form of an index linked to water use, and reported significant genetic variation for both leaf water potential and soil moisture availability under drought conditions (Gutierrez *et al.*, 2010). Building on these approaches, multispectral imaging enables the calculation of VIs using specific wavelengths that can be indicative of canopy biochemical composition. For instance, reflectance in the red-edge region (∼705–740 nm) is sensitive to chlorophyll content in sorghum, which can provide insights into photosynthetic capacity and nitrogen status (Li *et al.*, 2018). Additionally, Féret *et al.* (2021) showed that leaf protein content could be estimated using two narrow spectral domains between 2100–2139 nm and 2160–2179 nm, highlighting the potential of shortwave infrared regions to capture nitrogen-containing compounds. These examples underscore how specific spectral regions can be linked to canopy biochemical composition. Considering the above foundational studies, the empirical machine learning approach could utilize canopy temperature and VIs to predict RSA traits. If successful, this could offer advantages for more accurate selection or genetic dissection of RSA-specific genetic controls, as predicted trait values may be less confounded with phenology or other canopy traits across a breadth of genetic diversity.

This study explored the potential of UAV-captured VIs to predict RSA traits and above-ground biomass in barley. We trained machine learning models on ground-based phenotypes for a representative subset to evaluate prediction accuracy for both RSA and canopy traits. To demonstrate the potential to scale up the approach, the most accurate model was applied to predict the traits in the broader untested panel, which were then used for haplotype mapping to identify genomic regions associated with above- and below-ground traits. Our hypothesis is that the biochemical composition of the canopy at key growth stages captured by multispectral imaging can reflect genetic differences in root distribution, supporting the use of UAV-derived VIs to indirectly predict RSA traits. This research highlights UAV technology as a scalable solution for field-based root phenotyping, providing a pathway to develop climate-resilient barley varieties and the potential to extend these applications across diverse crops.

## Materials and methods

### Plant material

This study evaluated a panel of 544 diverse barley genotypes provided by InterGrain Pty. Ltd across two field seasons (2022 and 2024), including 102 genotypes tested in both years. The panel represents a broad spectrum of genetic diversity relevant to Australian barley breeding, which includes historic and modern breeding lines and commercial cultivars. The panel was genotyped using the Infinium™ Wheat Barley 40 K v1.0 BeadChip (Keeble-Gagnère *et al.*, 2021), yielding 12 561 high-quality single nucleotide polymorphism (SNP) markers. In both years, a diverse subset of 20 genotypes (Core20) was selected for intensive ground-based measurements and was used to generate the phenotypic data required for training machine learning models. The Core20 subset was carefully selected to minimize variation in flowering (∼5 d window), ensuring material was suitable for detailed root phenotyping across both years with minimal confounding effects of phenology. Importantly, Core20 was also representative of the genetic diversity in the broader panel (97% genetic coverage).

### Field trial locations and experimental designs

Field trials were conducted in Gatton, QLD, Australia. The 2022 experiments were located at 27°32′40.0′′S, 152°21′28.2′′E, while the 2024 experiments were conducted 3 km away from the 2022 site at 27°32′53.8′′S, 152°19′36.9′′E. Both sites are characterized by deep clay soils with a high water-holding capacity (Powell, 1982). All trials were sown at a target density of 120 plants m^–2^. Each season, two adjacent field experiments were conducted, a ‘Yield Trial’ and a root phenotyping trial ‘Coring Trial’.

In 2022, the Yield Trial comprised 600 plots using a randomized incomplete block experimental design optimized with the Optimal Design package V1.0.1 (Harman and Filova, 2019). Genotype allocation across rows and columns was guided by the additive realized relationship matrix calculated in the ASRGenomics R package (Gezan *et al.*, 2022) and partial replication of 1.5 replicates (Butler *et al.*, 2017; Filov and Harman, 2019).

In 2024, the Yield Trial consisted of 340 experimental plots, designed with the same plot dimensions and layout principles as the 2022 Yield Trial, and 1.3 replicates. The ‘Coring Trial’ in both years included the Core20 panel and contained plots with the same dimensions and seeding rate as the Yield Trial. These experiments adopted a randomized complete block design with six replicates per line. Allocation to row and column positions was optimized based on the additive realized relationship between individuals.

### Environment characterization of trial sites

To characterize the environmental conditions across seasons, trials were simulated using commercial cultivar RGT Planet with the APSIM-Barley crop model (version 2024.6.7514.0; Holzworth *et al.*, 2014, 2018). Simulations used weather data from the nearest meteorological station from the SILO-based dataset (https://www.longpaddock.qld.gov.au/silo/; Jeffrey *et al.*, 2001), soil information from a nearby soil characterized in the APSoil database (http://www.apsim.info/Products/APSoil.aspx), initial soil moisture at different depths measured onsite at sowing, and the trial management practices. To best simulate the growth and development of the crop in each trial, an APSIM parameter of cultivar RGT Planet (‘minLN’, i.e. the number of leaves that the crop produces under a long photoperiod when vernalization is satisfied early in the crop duration) was tuned so that simulated flowering time matched observed flowering time (Amin *et al.*, 2025, Preprint). Plant-available water at different soil depths was simulated for the dates when soil core measurements were taken.

### Above- and below-ground phenotypes for the Core20 panel

**Fig. 1.**
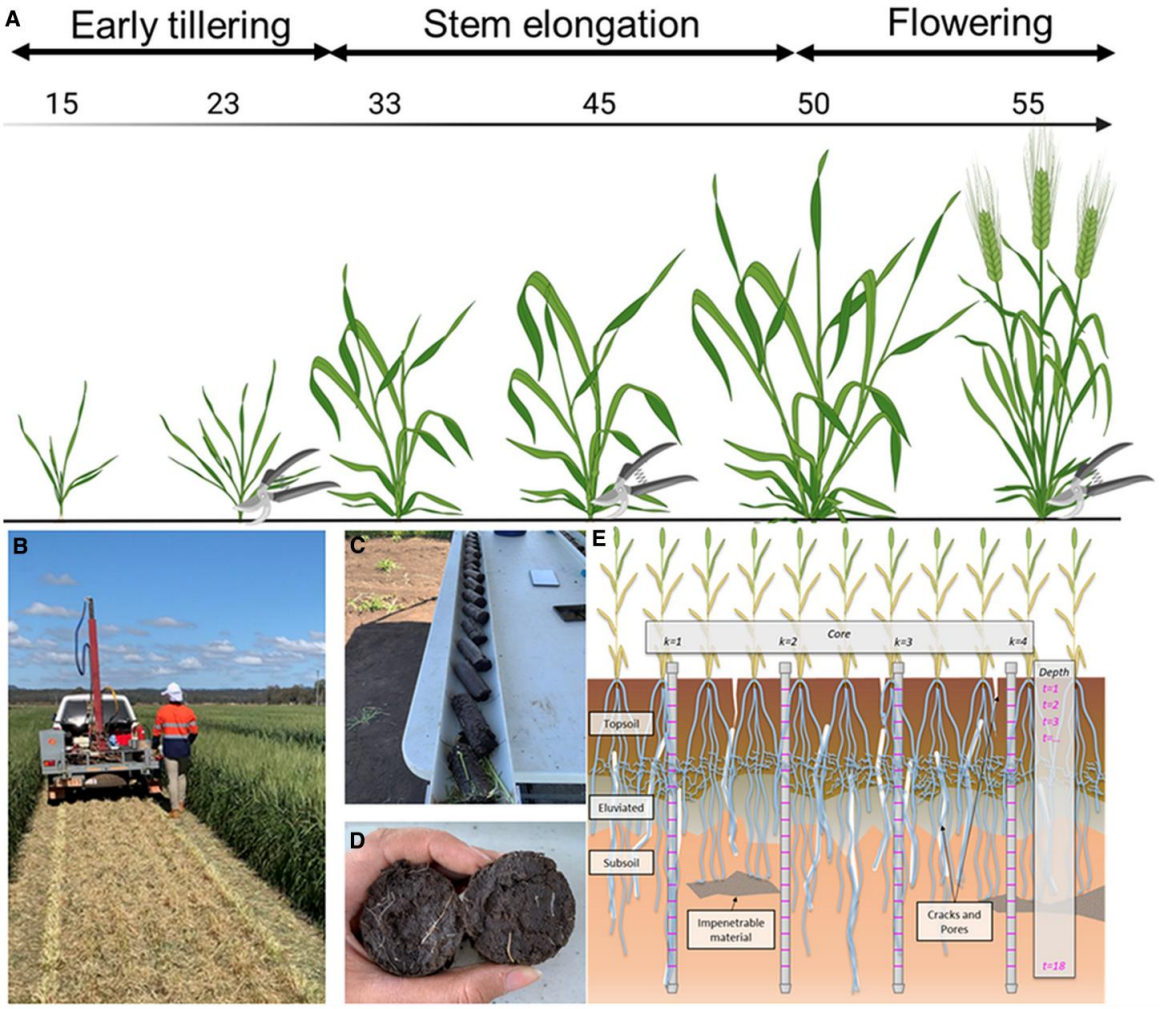
Overview of the ground-based measurements for above-ground dry biomass and below-ground root count in the coring trial. (A) Biomass sampling time points at the three key growth stages: tillering, stem elongation, and flowering time. Additional sampling time points at grain fill and maturity were collected in 2024. (B) Mulched plots at flowering time in preparation for soil coring using a rig mounted at the back of a trailer. (C) Breaking of soil cores into 10 cm intervals. (D) Visual counting of roots on each surface of the break. (E) Illustration of the position of the four cores sampled within each plot showing the inter- and intra-row sampling of the core break method as described by Wasson et al. (2014). BioRender.com was used to create Figure 1A.

The Core20 genotypes were phenotyped using ground-based measurements to assess above-ground biomass and below-ground root distribution. Above-ground biomass was measured at three time points corresponding to key growth stages (GSs) depending on the genotype: early tillering (between GS21 and GS25), stem elongation (GS30–GS39), and flowering time (GS50–GS59; Fig. 1). In 2024, two additional biomass sampling time points were collected at grain fill (GS80) and maturity (GS90) to support calibration of biomass prediction models. Plants within a 0.25 m^2^ quadrat were hand-harvested at ground level using a sickle, dried at 65 °C for 7 d, and above-ground dry biomass (SDB) was recorded.

The below-ground root phenotyping was performed at flowering time (GS50–GS59) using the previously reported ‘core break’ method (Wasson *et al.*, 2014). To accommodate this method, the entire canopy was mulched immediately prior to soil coring. A total of 480 soil cores were extracted per season, with four cores taken per plot, two each from the inter- and intra-rows of plants, avoiding the bordering plants to minimize edge effects. Once the cores were extracted from the ground, they were unloaded and broken into 10 cm intervals (from 0 cm to 170 cm), and a visual count of the number of roots visible on each surface of the break was recorded. The core break method has been successfully applied in wheat; however, this study marks the first time it has been applied in barley under field conditions. A subsample (10%) of the soil cores for two Australian commercial barley cultivars (RGT Planet and Maximus CL), which contrast for flowering time and phenology, was selected in 2022 for root washing to verify the association between root count and root dry biomass (RDB). RDB was recorded after drying at 65 °C for 7 d. A strong correlation between root count and RDB (*r*=0.63, *P*<0.001) confirmed the effectiveness of the ‘core break’ method in barley (Supplementary Fig. S1).

### UAV phenotyping

To investigate the potential of using UAV-captured VIs as proxies for canopy biomass and root system variation, UAV flights were performed across both field seasons (2022 and 2024) following consistent protocols. In 2022, intensive UAV phenotyping was conducted, with 17 flights performed from sowing to physiological maturity. In contrast, a more targeted approach was taken in 2024, with six UAV flights conducted at key developmental stages to capture phenotypic variation efficiently. Flight route, image capture, image processing, and VI extraction were performed as described by Smith *et al.* (2024). Briefly, all flights were performed using a Matrice 300 RTK-DJI drone fitted with a ‘MicaSense Altum’ multispectral and thermal high-resolution camera.

Flight altitude was set at 20 m with 80% side and front image overlap to ensure sufficient common tie-points during orthomosaic generation. These flight parameters resulted in a ground sample area of 0.86 cm^2^ and 13.5 cm^2^ per pixel for multispectral and thermal, respectively. Flights occurred on clear, still days between 10.00 h and 12.00 h, aligning with the ‘When2Fly’ application to minimize glare and enhance image resolution (Jafarbiglu and Pourreza, 2023). Further, 10 ground control points (GCPs) were placed in each experiment and their accurate GPS coordinates were recorded using Propeller Aeropoints (https://www.propelleraero.com/aeropoints/). The raw images were processed and stitched, using the GCPs to optimize image alignment in Agisoft Metashape software (Agisoft LLC, St. Petersburg, Russia). A single geo-referenced orthomosaic TIF image was generated per flight, consisting of six channels, namely blue (476±32 nm), green (560±27 nm), red (668±14 nm), red-edge (717±12 nm), near-infrared (842±57 nm), and long wave thermal infrared (LWIR) (11±6 µm). Shapefiles containing single plot polygons were created in ArcMap V10.8 to extract plot-level VIs.

Masked and unmasked VIs were calculated for each plot. Masked VIs were calculated by first deriving the Optimized Soil Adjusted Vegetation Index (OSAVI) and applying Otsu thresholding to the OSAVI-captured pixel values. This method enables the segmentation of green vegetation from soil pixels (Otsu, 1979), allowing the mean VI to be calculated exclusively from vegetation pixels within the plot. Unmasked VIs were calculated as the mean VI value across the entire plot, including both canopy and soil pixels. All raster calculations and zonal statistics were performed using a Python-based in-house package called Xtractori by researchers from the University of Queensland (Das *et al.*, 2022). This tool efficiently extracts a diverse range of VIs and canopy temperature (CT) traits from orthomosaics, with the flexibility to apply soil background masking depending on the target trait. For this study, a total of 60 spectral VIs (Supplementary Table S1) were calculated as described by Smith *et al.* (2024) in addition to three temperature-related indices.

### Data analysis

Phenotype data, including SDB at each time point and plot-level root counts across both years, were analysed using ASReml-R Version 4.2 (Butler *et al.*, 2017) in R version 4.3.0 (R Core Team, 2023). A linear mixed model (LMM) framework was employed to partition various components and account for spatial variation in the field. The model was fitted with genotype as fixed effect, while replicates were fitted as random effects. Best linear unbiased estimates (BLUEs) for adjusted genotype mean values were calculated using Equation (1):


(1) yijkm=μ+Repm+Rj+Ck+Gi(m)+eijk(m)

where *y_ijkm_* denotes the plot observation for genotype *i* in replicate *m*, row *j*, and column *k*. Fixed effects include the overall mean μ and genotype *G_i_*_(*m*)_, while random effects accounted for replicate Rep_m_, row *R_j_*, and column *C_k_*. The residuals *e_ijk_*_(*m*)_ were spatially correlated, following $N(0,AR1⊗AR1\sigma^{2}$), while the variance components for rows and columns were modelled as $R_{j}∼N(0,\;\sigma_{r}^{2}$) and $C_{k}∼N(0,\;\sigma_{k}^{2})$, respectively. The adjusted BLUEs were used for subsequent analyses and model training. The broad-sense heritability (*H*^2^) for root and canopy traits was calculated separately for each year.

**Fig. 2.**
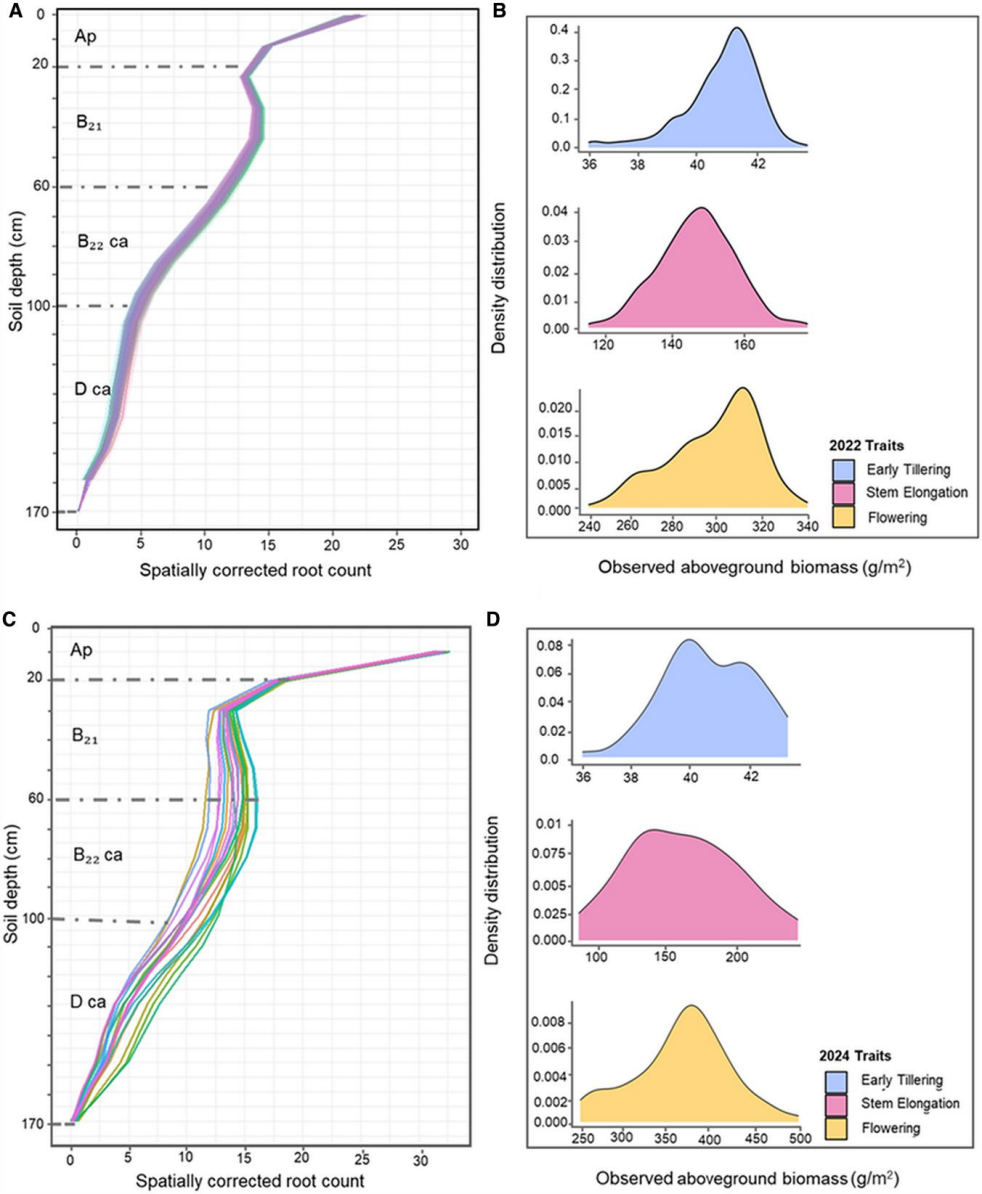
Root distribution and above-ground biomass for the Core20 genotypes used as a training dataset for phenotypic predictions. (A) and (B) correspond to the 2022 field trial, while (C) and (D) represent the 2024 field trial. (A, C) Spatially corrected root count for the different soil depths measured at flowering time, fitted by a spline-model; horizons from the soil are presented (Powell, 1982): 0–20 cm (Ap horizon, brownish-black clay, pH 6.5–7.8), 20–60 cm (B21 horizon, brownish-black heavy clay, pH 8.2–8.8), 60–95 cm (B22 ca horizon, brown clay with carbonate, pH 8.2–8.8), and 95–150 cm (D ca horizon, brown light clay with carbonate, pH 8.2–8.8). (B, D) Density distributions of above-ground biomass (g m^–2^) at three growth stages: early tillering (top), stem elongation (middle), and flowering (bottom).

A semi two-stage modelling approach was performed to gain insights into variation for the overall root system size and distribution at different depths to model root count over depth on a plot level (Pérez-Valencia *et al.*, 2022). Packages used as part of this modelling approach included the Spatial Analysis of Field Trials with Splines (SpATS) package (Rodriguez-Alvarez *et al.*, 2018) in combination with the statgenHTP package (Millet *et al.*, 2021) in R. Plot-level root count BLUEs were used for modelling longitudinal trends using a flexible hierarchical P-spline for both years. This modelling approach allows the calculation of five root proxy traits: (i) area under the root count curve, which is a proxy for overall RSA system size, and (ii–v) the area under the curve (AUC) at specific depths, namely 0–20, 20–60, 60–100, and 100–170 cm, as proxies for root distribution in different soil strata (Fig. 2A, C). Those five root proxies were used in the subsequent machine learning model training and spatial analysis.

### Training datasets

Three datasets were used to train predictive models: (i) high-quality ground-based measurements for above-ground biomass collected on the Core20 panel at three key growth stages in 2022 (i.e. early tillering, stem elongation, and flowering time) and five stages in 2024, including additional sampling time points at grain fill and maturity, (ii) precise below-ground root counts for Core20 using the ‘core break’ method, and (iii) UAV-calculated VIs for both the Core20 and the broader panel derived from 17 UAV flights in 2022 and six flights in 2024. Using these datasets, we investigated whether UAV-captured VIs could predict both SDB and RSA traits (specifically, the AUC for root count at various depths) across the broader panel of 544 genotypes tested across both seasons. The methodology is detailed below.

### Canopy biomass prediction models

Raw SDB values measured at key developmental stages were assembled alongside the corresponding VIs measured from UAV images captured simultaneously. In 2022, SDBs from three time points were captured, whereas five were sampled and used for predictions in 2024, three of which are displayed for consistency (Fig. 2B, D). Subsequently, the data frame (165 SDB observations in 2022 and 314 observations in 2024, and 63 predictor VI variables) was split into train and test sets using an 80%/20% split. All model development and validation steps were conducted independently for each season. Sampling was stratified based on the timing of SDB measurements to ensure even sampling across the growth stages.

This study employed two commonly used machine learning approaches to predict SDB, namely PLSR and RF using the Caret package in R (Kuhn, 2008). The first model, PLSR baseline, containing all 63 variables (PLSR_allvars) was used to identify key variables associated with SDB. This model used leave one out cross-validation and a tuning grid of 1:63 for the parameter ‘ncomp’, which defines the number of components in the model. The optimal number of components was chosen based on the minimum root mean square error (RMSE) achieved across the cross-validation. Subsequently, the VarImp function from the Caret package was used to rank all input variables in PLSR_allvars (Supplementary Table S1), achieved by taking the weighted sums of variable coefficients across all relevant components (Kuhn, 2008). Starting from the top-ranked variable, we calculated Pearson’s correlation (*r*) between each variable and the next-best-ranked variable. If the correlation was less than a threshold of *r*=0.8, that variable was also added to the list of final variables for each trait and referred to as ‘topvars’. The number of topvars chosen was based on the ‘elbow method’, which iteratively increases the number of variables in a simple multiple linear regression model until a point of inflection in the model variance, which signifies the point of diminishing returns in performance related to the inclusion of any additional variable (Zhang, 2022).

Subsequently, another PLSR model (PLSR_topvars) was created using the top variables to compare model prediction accuracy before and after the variable selection process. In addition to PLSR, we also trained two separate models using RF to estimate SDB. A baseline RF model (Ranger_allvars) was first trained using the ‘Ranger’ package in R, using all 63 variables. Hyperparameter tuning involved iterating through the number of randomly selected features in the model; ‘Split.Rule’ was set to variance, the min.node size was set to 5, and the number of trees was set to 150. A refined RF (Ranger_topvars) was also trained using the top variables selected using the PLSR_allvars. The optimal number of components was chosen based on the maximum coefficient of determination (*R*^2^) and minimum RMSE, and relative (rRMSE) achieved across the cross-validation as detailed in Equations (2)–(4) below:


(2) $$R^{2}=1\text{–}\frac{\sum {\left(y_{i}\;\text{–}\;{\hat{y}}_{i}\right)}^{2}}{\sum {\left(y_{i}\;\text{–}\;\bar{y}\right)}^{2}}$$

(3) $$\mathrm{RMSE}=\sqrt{\frac{1}{n}\sum_{i\;=\;1}^{n}\;{\left(y_{i}\;\text{–}\;{\hat{y}}_{i}\right)}^{2}}$$

(4) $$\mathrm{rRMSE}(\text{\%})=\frac{\mathrm{RMSE}}{\bar{y}}\times 100$$

where *y_i_* is the observed value for the *i*th observation, ${\hat{y}}_{i}$ is the predicted value for the *i*th observation, *n* is the total number of observations, and $\bar{y}$ is the mean value of all observations.

To evaluate the model performance, *R*^2^, RMSE, and rRMSE were used. Both training and test datasets were utilized to evaluate model performance across both years and developmental stages. Spatial analysis using the ASReml-R V4.2 was conducted to assess the spatial variability of predicted biomass values and to estimate variance components and heritability of biomass sampled across the untested panel in the Yield Trial.

### Root system architecture prediction model

RSA traits, including the total AUC of the root count curve and AUC for specific soil depths (i.e. 0–20, 20–60, 60–100, and 100–170 cm), were also estimated using the four previous modelling approaches. These depth intervals were selected to align with the major soil horizons at the experimental site, as described by Powell (1982), which reflect key changes in soil texture and chemical properties relevant to root growth: 0–20 cm (Ap horizon), 20–60 cm (B21 horizon), 60–95 cm (B22 ca horizon), and 95–150 cm (Dca horizon). Unlike the biomass prediction models, which utilized VIs corresponding to biomass cut dates, the RSA prediction models used VIs collected throughout the entire growing season. Due to differences in flight frequency, separate models were developed for each season (2022 and 2024). This resulted in datasets including 550 RSA trait observations (5 traits × 110 plots) in 2022 and 600 RSA trait observations (5 traits × 120 plots) in 2024, each with 1071 and 378 predictor variables, respectively. The PLSR_topvar model demonstrated the best performance and was used to predict RSA traits for the untested panel in the Yield Trial each year. Spatial analysis using ASReml-R V4.2 enabled variance partitioning and heritability estimation for each RSA trait within each environment.

### Genome-wide haplotype mapping using predicted canopy and root system architecture traits

A total of 12 561 SNP markers were subjected to quality control, and full marker profiles were provided following imputation using Beagle version 5.4 software (Ayres *et al.*, 2012). This included the removal of markers with minor allele frequency (≤0.05) and those with >10% heterozygosity, resulting in 6451 high-quality polymorphic SNPs (Gower, 1966; Murtagh and Legendre, 2014). Following marker curation, population structure analysis was conducted using the ‘SelectionTools’ package in R. Genetic distances between individuals were calculated using modified Roger’s Distance (Rogers, 1972). Classical multi-dimensional scaling based on k-means clustering was applied to the data, followed by hierarchical clustering using Ward’s method (‘Ward.D2’) to minimize within-cluster variance (Murtagh and Legendre, 2014).

The local genomic estimated breeding value (LGEBV) haplotype mapping approach was implemented as per Voss-Fels *et al.* (2019). Phenotypic data used in this approach were BLUEs calculated for the five predicted RSA traits and the three canopy SDB values using the PLSR_topvar model applied to the broader panel of 544 genotypes tested across both years. Linkage disequilibrium (LD) blocks were assigned using the ‘Selection Tools’ package, based on patterns of LD present in the population. A total of 2451 LD blocks were classified by applying an LD threshold of 0.7 and a marker tolerance of 3, which resulted in an average of 2.6 markers per block. For each haplotype, LGEBV was calculated by first determining the individual SNP effects using a ridge-regression best linear unbiased prediction model (rrBLUP). Then the SNP effects within each haplotype were summed to provide the haplotype effect. The variance for haplotype effects within each LD block was calculated, and haploblocks with the highest variance were selected using a scaled variance threshold of 0.2, representing the top-performing haploblocks for RSA traits and SDB (top 0.08–0.82% of blocks across the traits). To visualize the most important blocks for SDB and RSA traits, we used CMplot version 4.5.1 (Yin, 2023). The physical start and end positions of the top 0.2% high-variance haploblocks were determined, along with previously reported QTLs, and known genes influencing above- and below-ground traits in barley (using Morex V1 as a reference) were plotted in MapChart (Voorrips, 2002) for visual comparison (Supplementary Fig. S2).

## Results

### Variation for root and canopy traits in contrasting environments

The Core20 panel, a diverse subset of 20 genotypes, was subjected to ground-based phenotyping for above-ground biomass and below-ground root distribution, providing training datasets for machine learning models to predict these traits in the larger set of 394 breeding lines in 2022 (Fig. 2A, B) and 252 lines in 2024 (Fig. 2C, D). SDB measurements collected for the Core20 subset at early tillering, stem elongation, and flowering time in 2022 displayed a consistent SDB increase over time, with a substantial range observed across the three time points. For instance, the adjusted mean value for SDB at early tillering was 37.0 g m^–2^, with a range spanning from 24.5 g m^–2^ to 49.0 g m^–2^. For SDB during stem elongation, the adjusted mean value was 129.9 g m^–2^ with a range spanning from 74.9 g m^–2^ to 215.6 g m^–2^, and the SDB adjusted mean value at flowering time was 305.6 g m^–2^ with a range of 191.8 g m^–2^ to 425.0 g m^–2^ (Fig. 2B). In 2024, five biomass sampling time points were taken to capture key growth stages, with adjusted mean SDB values increasing steadily from early tillering (29.1 g m^–2^; range 16.2–37.1 g m^–2^) to stem elongation (162.2 g m^–2^; range 97.3–235.2 g m^–2^), flowering (443.5 g m^–2^; range 260.4–549.2 g m^–2^), grain filling (532.3 g m^–2^; range 310.3–821.4 g m^–2^), and maturity (578.7 g m^–2^; range 377.2–795.8 g m^–2^; Fig. 2D).

Plot-level root counts showed considerable variability for root distribution across different soil strata and seasons, with a progressive decrease in root counts observed from the upper to the deeper layers of the soil (Supplementary Fig. S3A, B). In 2022, the mean root count at 10 cm depth was 23.2, gradually decreasing to 0.4 at 170 cm depth. Genotypic variation in root distribution was observed within each root section, with greater variability observed within the upper layer of the soil depth. For instance, root count at 20–60 cm ranged from 5 to 38.5. In 2022, plot-level variation in root counts was significant from 0 cm to 60 cm, whereas in 2024, this significance extended much deeper in the soil, up to 120 cm. The 2024 season also exhibited a higher overall root count compared with 2022, probably a result of the drier soil environment. For instance, the average root count at 10 cm depth was 32.9, decreasing to 0.27 at 170 cm depth. The highest variation for root count was observed between 30 cm and 130 cm, where the 2024 soil environment probably accentuated genotypic differences in root development.

Five root proxy traits were calculated from modelling the root over depth, including the total AUC of the root count curve and the AUC of the root count curve at various soil depths (Fig. 2A), and significant genotypic variation was observed. For instance, for the AUC at the 0–20 cm depth interval, values ranged from 17.7 to 19.9, with an average of 18.2. In the deeper sections of the soil, the AUC increased significantly, with the highest average AUC observed in the 20–60 cm depth (41.1). However, the AUC for 100–170 cm soil depth experienced a dramatic decrease in root growth and distribution compared with the other RSA traits. The overall AUC value, representing the cumulative root activity across all depth intervals, ranged from 90.6 to 192.9, with an average of 128.1 (Fig. 2A). In contrast, the five 2024 root proxy traits also showed substantial variation: the total AUC averaged 1668 (range 1141–2330); for the 0–20 cm depth, AUC averaged 247 (range 238–300); for 20–60 cm, the average was 566 (range 446–714); for 60–100 cm, the average was 516 (range 325–734); and for 100–170 cm, the average was 345 (range 123–763; Fig. 2C).

### UAV-captured vegetation indices enable the prediction of shoot biomass at key growth stages

UAV-captured VIs accurately predicted SDB across three growth stages for 2022 and five stages for 2024 in the Core20 panel cused for model training (Supplementary Fig. S4A, B), enabling the use of SDB data to train PLSR and RF models with 63 high-correlation (≥0.8) variables to improve biomass prediction accuracy. Key variables were identified through PLSR to reduce redundancy and maximize predictive power. A principal component analysis (PCA) was performed for each season, and this revealed that VIs captured across the different growth stages explained the majority of variation in canopy biomass. For instance, PC1 and PC2 explained 90.9% of the variation in 2022 (Supplementary Fig. S5A), while PC1 and PC2 explained 89.5% of the variation in 2024 (Supplementary Fig. S5B). VIs and related traits contributing strongly to variation in 2022 included: mean_r_red, Coverage_ndvi, osavimasked_mean_mtvi, mean_exg, Coverage_ndre_r, osavimasked_mean_temperature, and mean_msavi. In 2024, the most important contributors to model prediction ability were osavimasked_mean_ndvi_RedEdge, osavimasked_mean_temperature, and osavimasked_mean_ varigreen. Following this, refined PLSR and RF models were used, utilizing the top variables identified using the PLSR_allvars. Notably, variables such as Modified Soil-Adjusted Vegetation Index (MSAVI) and Osavi-masked mean temperature were key VIs for biomass prediction, and had a significant negative association with biomass at flowering time (*r*= −0.97, *r*= −0.72, respectively). A strong correlation between observed SDB and predicted SDB at each growth stage was found in both training and testing datasets using PLSR and RF models. However, the final model used for biomass prediction in the broader panel was the PLSR model with top variables, which demonstrated improved accuracy and consistent performance across both years (Supplementary Fig. S5A). For instance, in the training set for 2022, the model exhibited an RMSE of 22.6 g m^–2^, an rRMSE of 14%, and an *R*^2^ of 0.98; and for the independent testing set, an RMSE of 21.9 g m^–2^, rRMSE of 15.2%, and *R*^2^ of 0.98 (Supplementary Fig. S4A). Despite having fewer UAV flights in 2024, the RF model still achieved strong predictive performance, with an RMSE of 29.2 g m^–2^, rRMSE of 8.4%, and *R*^2^ of 0.99 in the training set and an RMSE of 63.2 g m^–2^, rRMSE of 18.7%, and *R*^2^ of 0.96 in the independent testing set (Supplementary Fig. S4B).

### UAV-captured vegetation indices enable the prediction of root traits in the field

**Fig. 3.**
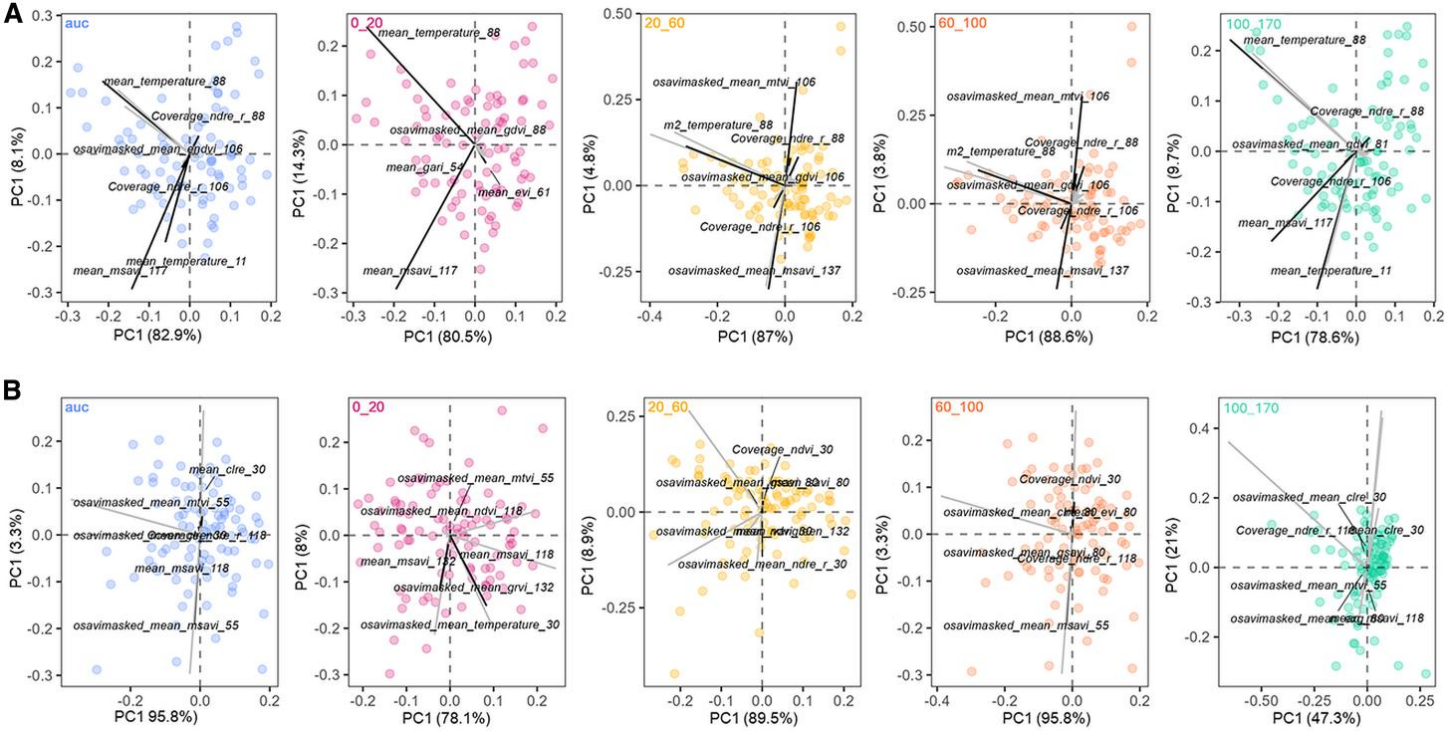
Biplots of vegetation indices (VIs). VI biplots used to predict root traits at various root count curves in 2022 (A) and 2024 (B). The set of biplots illustrates the principal component analysis (PCA) of the final vegetation indices used to predict the area under the root growth count curve (AUC) overall and at four specific depth intervals. Each biplot (left to right) represents AUC (overall), 0–20, 20–60, 60–100, and 100–170 cm.

**Fig. 4.**
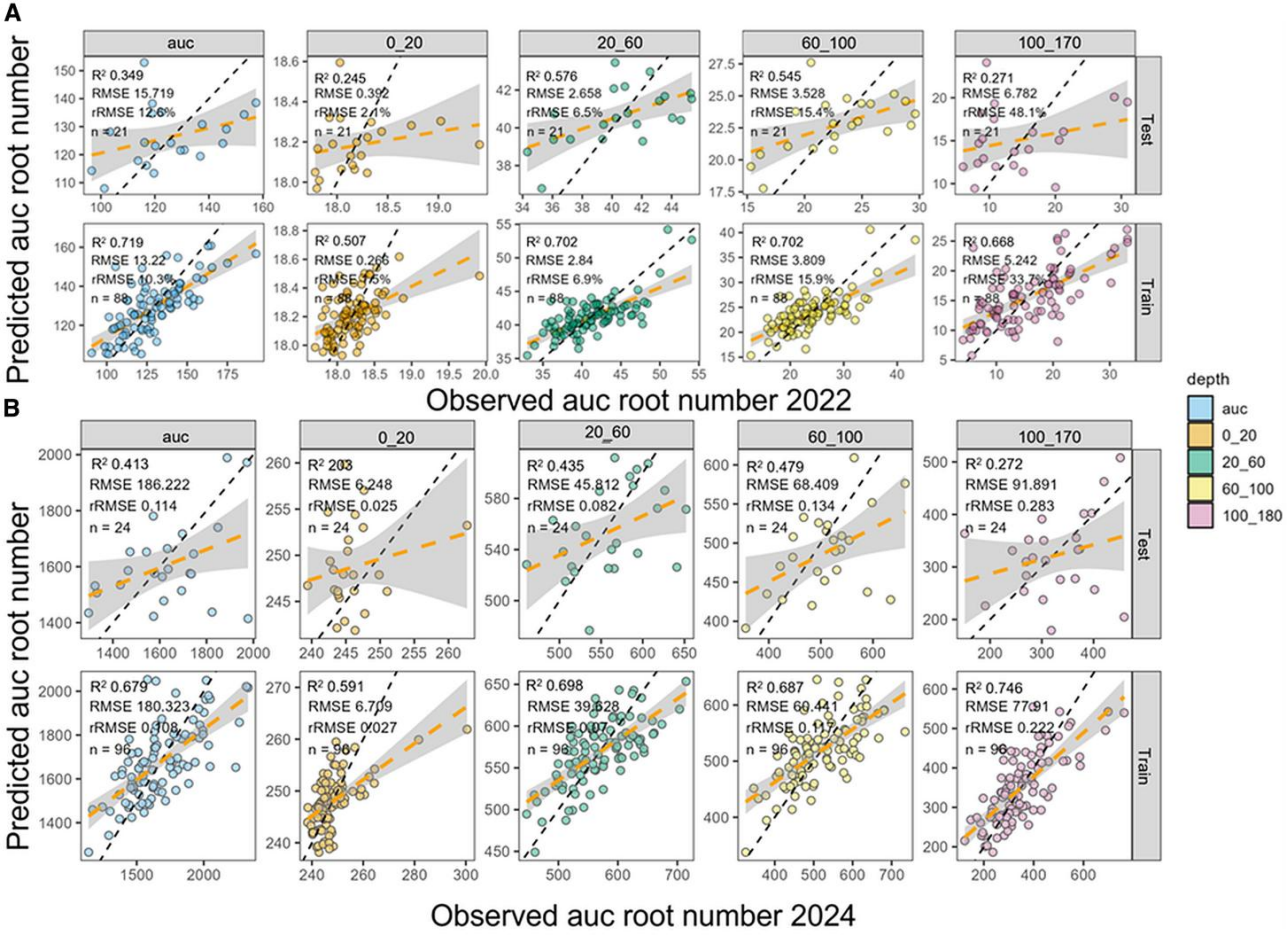
Observed versus predicted root traits for the Core20 genotypes using UAV-derived vegetation indexes. (A) 2022 predictions and (B) 2024 predictions using partial least square regression with the top-ranked variables (PLSR_topvars). The model was trained on 80% of the data and validated on the remaining 20%. Each panel shows model performance for a specific root trait: area under the curve for root count (AUC), and AUC for specific depth intervals, 0–20, 20–60, 60–100, and 100–170 cm).

UAV-captured VIs, initially utilized to predict SDB at three growth stages (early tillering, stem elongation, and flowering), were also employed to predict RSA traits (Fig. 2). These traits included the AUC of the root count curve and the AUC at specific depths corresponding to different soil strata (Fig. 2A, C). The final PLSR model using top variables identified through correlation filtering, and PCA was applied to predict RSA traits for 394 and 252 genotypes in 2022 and 2024, respectively. Using the top selected VIs by the PLSR model, PC1 explained 78.6–88.6% variation in 2022 and 47.3–95.8% in 2024, and PC2 explained 3.8–4.3% variation in 2022 and 3.3–21% for all RSA traits (Fig. 3A, B). Interestingly, flowering and early grain fill emerged as key growth stages where UAV-derived VIs were most predictive of root traits across depths and years. In particular, the normalized difference red-edge index (NDRE) captured at 88 days after sowing (DAS) was consistently important for predicting root traits in the 0–20, 20–60, and 60–100 cm layers across seasons (Fig. 3). Mean temperature at 88 DAS and mean_msavi_117 were also strong predictors of root size and distribution. In 2024, the overall AUC of the RSA trait was best predicted by indices such as mean_msavi_118, mean_clre_30, and mean_mtvi_55, suggesting that VIs captured during early tillering, stem elongation, and early grain fill stages were particularly informative. Indices such as msavi, mtvi, clre, and ndvi, captured ∼55–118 DAS, were frequently selected by the model and contributed to predicting root traits at both shallow and intermediate depths (Supplementary Table S1). Overall, the PLSR model trained on UAV-based VIs demonstrated robust and consistent performance across seasons, with enhanced accuracy observed particularly for traits measured in the upper (0–20 cm) and intermediate (20–60 cm) soil layers (Fig. 4). For 2022, RMSE ranged from 0.27 to 13.2, rRMSE from 1.5% to 33.7%, and *R*^2^ from 0.51 to 0.72 across different RSA traits. In 2024, model performance remained robust, with RMSE values ranging from 6.2 to 180.3, rRMSE from 2.7% to 22.2%, and *R*^2^ from 0.59 to 0.75. This confirms the model’s capacity to predict root traits with reasonable accuracy across contrasting environments (Fig. 4A, B).

Predicted RSA traits varied significantly across years, demonstrating a range of values for different RSA traits corresponding to the modelled overall AUC and the AUC at specific depths (Supplementary Table S2; Fig. 4). Broad-sense heritability (*H*^2^) for predicted root traits averaged 0.71 in 2022 and 0.81 in 2024 (Supplementary Table S2), indicating that a large proportion of the observed phenotypic variation was due to genetics in each year. The highest heritability values were observed in the shallow (0–20 cm) and intermediate (60–100 cm) soil layers in both seasons, reaching up to 0.90 in 2024. In contrast, the deepest layer (100–170 cm) exhibited lower heritability, probably reflecting increased environmental variability at depth. Interestingly, the heritability of overall root size (AUC) was notably higher in 2024 (*H*^2^=0.92) than in 2022 (*H*^2^=0.65).

### Predicted root and shoot traits vary under contrasting environmental conditions

Based on environment characterization in APSIM, the 2024 trial experienced greater water stress than the 2022 trial. The 2022 trial received substantially more in-season rainfall compared with the 2024 trial (Supplementary Fig. S6C, D), which resulted in higher plant-available water in the soil profile across key growth stages (Supplementary Fig. S6E, F). In addition, the 2024 trial also experienced higher maximum temperatures during the critical flowering and grain-filling periods (Supplementary Fig. S6A, B).

**Fig. 5.**
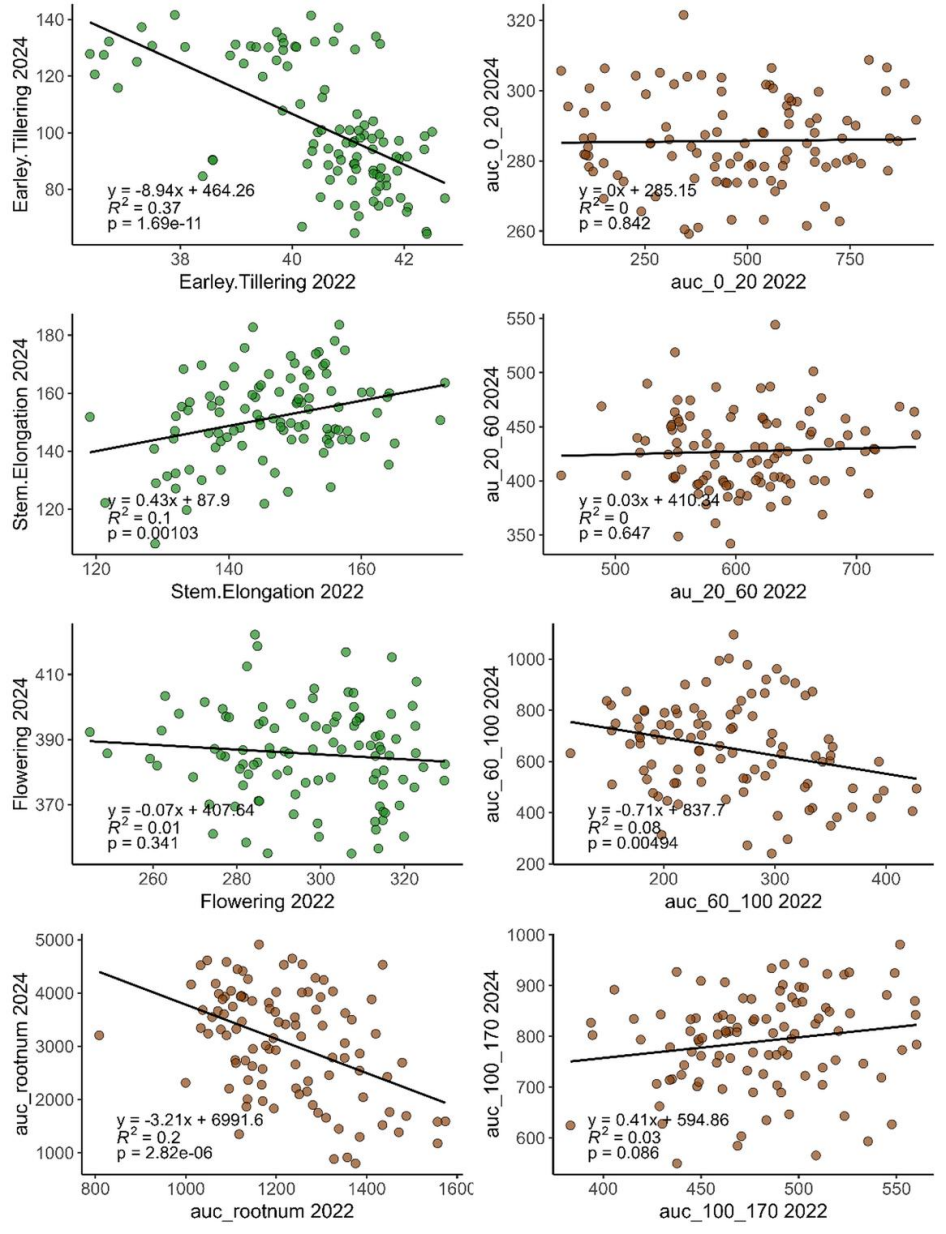
Cross-seasonal relationships between predicted shoot and root traits for 102 genotypes evaluated in the 2022 and 2024 field trials. Panels compare predicted above-ground biomass at early tillering, stem elongation, and flowering with predicted root system architecture traits, including overall root system size (AUC) and depth-specific traits (0–20, 20–60, 60–100, and 100–170 cm). All values are derived from UAV-based machine-learning models. Each scatterplot shows the fitted regression line, coefficient of determination (*R*^2^), and *P*-value.

We investigated the relationship between predicted above- and below-ground traits across the contrasting environments by comparing model outputs for the 102 barley genotypes in common across the 2022 and 2024 trials (Fig. 5). Above-ground biomass predictions were moderately consistent across years at early tillering (*R*^2^=0.37, *P*<0.001) and stem elongation (*R*^2^=0.10, *P*=0.001), while flowering biomass showed no significant correlation (*R*^2^=0.01). For below-ground traits, the overall AUC demonstrated moderate cross-seasonal correlation (*R*^2^=0.20, *P*<0.001), while depth-specific RSA traits showed little to no relationship across the two contrasting environments.

### Population structure of the barley panel

**Fig. 6.**
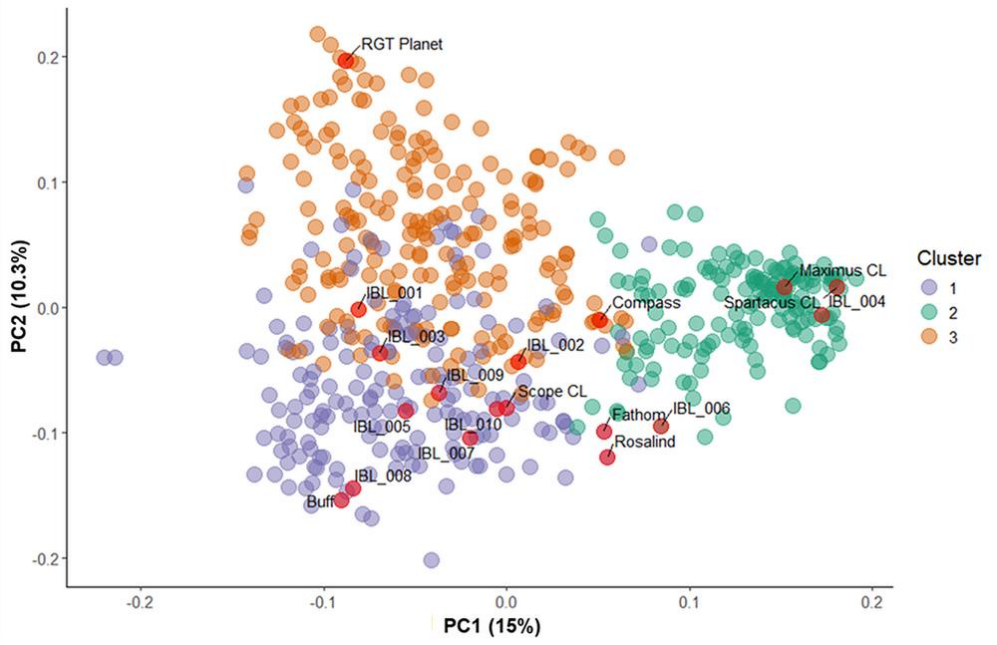
Population structure of the Australian barley breeding panel, calculated from 6451 polymorphic SNP markers for 544 lines. Three main clusters were identified: Cluster 1 consists of Australian germplasm pool 1, including germplasm from the VIC and SA breeding programmes. Cluster 2 represents European-derived barley breeding lines and varieties. Cluster 3 comprises Australian germplasm pool 2, which includes lines that are descendants of acid-tolerant material, generally originating from the historic WA and QLD breeding programmes. Genetic distance was calculated, with the variance explained by principal components being PC1=15% and PC2=10.3%. The subset of 20 genotypes (Core20) evaluated in the Coring Trial are presented in red and represent 97% of the Australian barley breeding panel.

To support haplotype mapping of predicted RSA and biomass traits, we examined the genetic structure of the barley panel. Clustering analyses identified three distinct hierarchical clusters. Cluster 1 represents an Australian germplasm pool 1 originating from ancestral south-eastern Australian breeding programmes, and includes modern varieties such as Spartacus CL, Maximus CL, and Compass. Cluster 2 consists of European-derived barley lines and varieties such as RGT Planet and Oxford. Cluster 3 encompasses a second Australian germplasm including the varieties Buff, Combat, and Scope CL, which are derived from either the ancestral Western Australian and Victorian barley breeding germplasm pool or the more recent InterGrain breeding outcomes. A PCA revealed that 62.01% of the total variance was captured across 10 PCs, with the first two PCs accounting for 15% and 10.3% of the variance, respectively (Fig. 6).

### Haplotype mapping reveals genomic regions associated with root and shoot traits

**Fig. 7.**
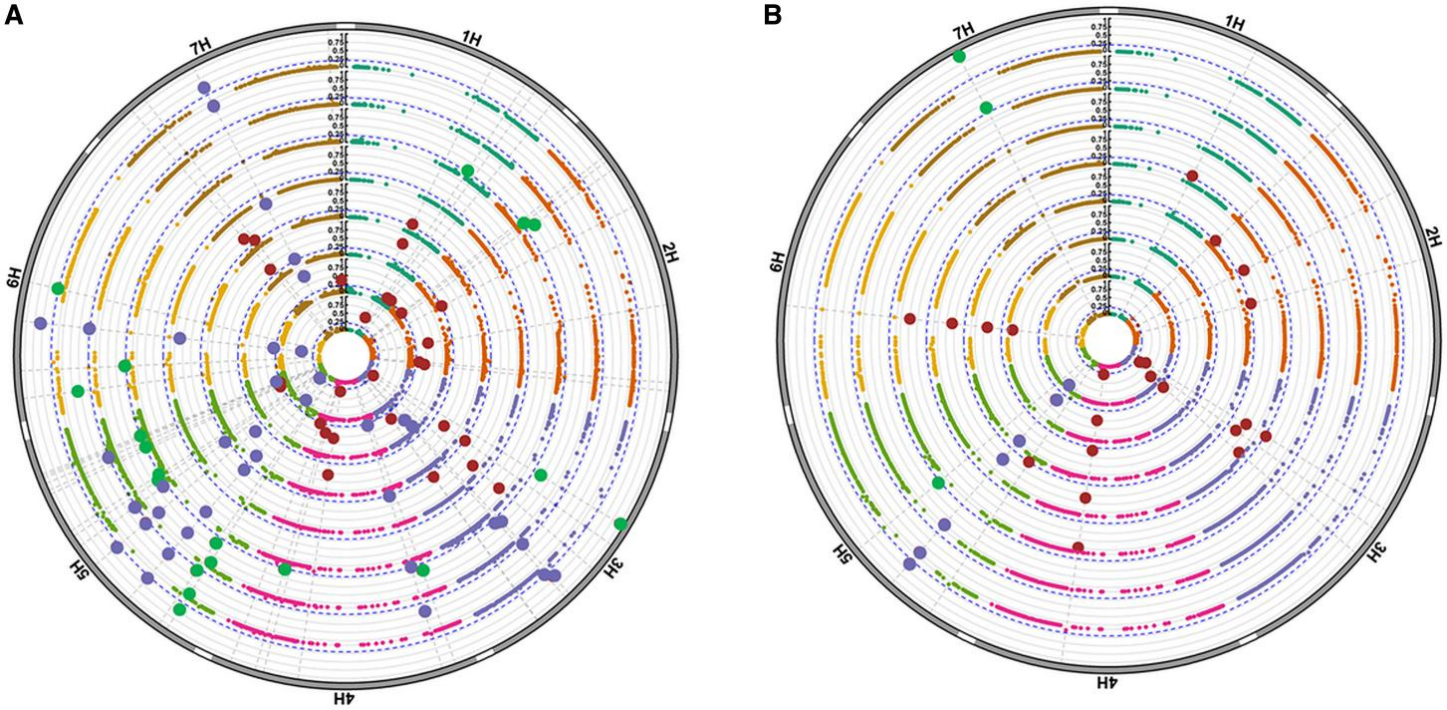
Circos Manhattan plot displaying top haploblocks associated with shoot and root traits in this study during (A) 2022 and (B) 2024. Concentric rings (inner ring—outwards) represent the area under the curve (AUC) for overall root count and at various soil depths, and above-ground dry biomass at key growth stages. Haploblocks are classified within each season according to those that are associated with root and shoot traits (blue), shoot traits only (green), and root traits only (brown). Barley chromosomes (1H–7H) are labelled on the outer circumference. Only the most highly associated haplo-blocks for each trait are presented (with a scaled variance ≥0.2). Chromosomal positions for haploblocks are provided in Supplementary Table S3.

A total of 47 haploblocks exceeding a scaled variance threshold of ≥0.2 were identified across the two contrasting seasons, revealing key genomic regions associated with root and shoot development (Fig. 7; Supplementary Table S3). Of these, 23 haploblocks were associated with RSA traits and 13 with shoot development. Notably, 11 haploblocks demonstrated pleiotropic effects on both root and shoot traits. As the predicted root and shoot traits varied in the contrasting environments and to further dissect environment-specific associations, classes of haploblocks were determined for each trial: ‘root-specific’ (detected as a top block for an RSA trait), ‘shoot-specific’ (detected as a top block for a canopy biomass trait), and ‘both root–shoot’ (detected as a top block for both a canopy biomass trait and an RSA trait).

In 2022, 45 haploblocks were significantly associated with RSA or SDB traits, including 21 root-specific, 13 shoot-specific, and 11 both root–shoot haploblocks. Among these, the b001081 haploblock on 4H emerged as a major contributor to root development, being consistently associated with total root size (AUC) and with RSA traits at 0–20 cm and 20–60 cm depths. Shoot-specific associations included b001336 (5H), which was linked to canopy biomass at early tillering and stem elongation stages. In 2024, a total of 13 haploblocks met the significance threshold, comprising 10 root-specific, two shoot-specific, and a single both root–shoot haploblock. Notably, b001340 (5H) exhibited the strongest pleiotropic signal, being associated with 14 traits across both seasons, spanning RSA and canopy biomass traits. This consistency across environments highlighted the importance of b001340 as a potential key regulator of root–shoot dynamics under varying field conditions.

### Alignment of haploblocks with previously reported quantitative trait loci and genes

Many of the haploblocks identified in this study aligned with previously reported QTLs and key developmental genes in barley, highlighting their potential roles in modulating canopy biomass and RSA traits (Fig. 7; Supplementary Table S4). In 2022, several shoot- and root-related haploblocks aligned with established loci and known genes. For instance, shoot-associated haploblocks included b000072 (*QRWC.1H*, 1H; Chen *et al.*, 2010), b000353 (*DRO1*, 2H; Uga *et al.*, 2011), and b000370 (*NRC162* and *NRC160*, 2H; Siddiqui *et al.*, 2024), all contributing to early tillering biomass. Further root and shoot associations in 2022 were also notable, including root-specific haploblock b001495 and shoot-specific haploblock b001502 (5H), which were in close proximity to the vernalization gene *VRN-B1* (Voss-Fels *et al.*, 2018), and b002248 (7H), aligned with *WSC10040* (Diab *et al.*, 2004), contributing to both shoot and root traits across multiple stages. The b001340 haploblock on 5H aligned with the *EGT2* gene (Kirschner *et al.*, 2021), known to control root gravitropism, and was associated with 14 traits across both years, including early tillering, stem elongation, flowering, and multiple root traits across 0–170 cm. Likewise, b000756 on chromosome 3H overlapped with *RAQ1* (Robinson *et al.*, 2016) and was consistently associated with root biomass at depth (60–100 cm and 100–170 cm), as well as early tillering and flowering biomass.

In 2024, 13 haploblocks were identified, of which several aligned with known genes. For instance, b000240 (2H) was associated with *PPD-H1* (Campoli *et al.*, 2012), and b002248 (7H) continued to show associations with *WSC10040* across growth stages. The co-location of haploblocks with major genes such as *DRO1*, *EGT2*, and *PPD-H1*, and the close proximity to *VRN-B1* highlight the accuracy of predicted RSA and canopy traits derived from UAV phenotyping. This also underlines the robustness of the mapping strategy in identifying genomic regions that control root and shoot trait variation across contrasting environments.

## Discussion

This study extends the utility of UAV phenotyping beyond traditional canopy trait analysis by using a large number of UAV-captured VIs, in combination with machine learning algorithms, to predict below-ground root traits. Earlier studies have demonstrated that UAV phenotyping can predict above-ground biomass and water-use efficiency in wheat and sorghum. These studies used single VIs, such as MSAVI or leaf area index, as indirect proxies for soil water available to the crop (Lopes and Reynolds, 2010; Pinto and Reynolds, 2015; Potgieter *et al.*, 2017). Here, we initially explored 60 spectral VIs and three canopy temperature-related traits across different growth stages; however, only the most informative indices contributing to model accuracy were used to predict RSA traits (Fig. 3). Interestingly, the most predictive VIs varied between seasons, highlighting how contrasting environmental conditions, such as water availability in the drier 2024 season, can influence which spectral signals are most relevant for capturing below-ground variation (Supplementary Fig. S6). By integrating machine learning with ground-truth RSA training datasets, we have developed a scalable solution to phenotype RSA and canopy traits simultaneously, which has the potential to advance research and help address the bottleneck of evaluating large populations under field conditions.

Substantial variation in canopy biomass and RSA traits was observed across the barley panel, providing a strong foundation for genetic analysis. The high heritability observed for predicted root and shoot traits across both seasons highlights the robustness of UAV-based phenotyping and machine learning approaches for dissecting complex traits in barley (Supplementary Table S1). Notably, accurate predictions were achieved in 2024 despite using only five UAV flights, demonstrating the efficiency and scalability of this approach. Additionally, the drier environmental conditions in 2024 appeared to enhance differentiation among genotypes for RSA traits, providing greater contrast for exploring genetic architecture or RSA (Fig. 2C). This is particularly important when targeting breeding for root traits under drought (Siddiqui *et al.*, 2024). Targeting variation for RSA is also critical for resource acquisition. For example, the concentration of roots in the upper soil layers observed in the 2022 wet season aligns with previous findings linking nutrient availability to root density. These findings complement prior research in wheat that connected RSA traits to enhanced water uptake and decreased canopy temperature using UAV phenotyping technology (Lopes and Reynolds, 2010). Furthermore, the observed variation in above- and below-ground traits among the Core20 subset was important for training and testing machine learning models to ensure robust model development for accurate trait prediction of the broader panel across seasons.

While the predicted proxy for root system size (AUC) showed a moderate correlation across the wet (2022) and dry (2024) environments, there was no relationship between the root traits at specific depths across the environments (Fig. 5). These results highlight the plastic nature of root systems and their responsiveness to environmental conditions, such as differences in soil moisture, temperature, and timing of rainfall. While barley genotypes may have some degree of genetic predisposition for root system size, the distribution of roots appears to be highly influenced by the weather conditions in-season. Future research should integrate field experiments with modelling approaches (Manschadi *et al.*, 2006; Rich *et al.*, 2016) to validate the value of specific RSA traits under different production scenarios. This could confirm whether deeper roots enhance drought resilience or shallow roots improve nutrient uptake (Henry *et al.*, 2010; Lynch, 2019), strengthening the case for including these traits in breeding programmes targeting environmental adaptation.

Haplotype mapping using predicted traits for the 544 breeding lines further demonstrated the utility and value of this approach by enabling the identification of genomic regions associated with root and shoot traits under field conditions. A total of 47 haploblock–trait associations were detected across the two seasons. Of these, 34 were linked to root traits and 24 to shoot traits, highlighting the complex and variable genetic control of the traits across growth stages and environments (Fig. 7). For example, haploblock b002248 (7H), which was associated with canopy biomass traits in our study, co-located with *WSC10040*, a water-soluble carbohydrate QTL previously reported under drought stress (Diab *et al.*, 2004). Similarly, b000353 (2H) overlapped with the *DRO1* gene, a key regulator of root angle and drought avoidance (Uga *et al.*, 2011), and was associated with canopy biomass at early tillering, reinforcing its relevance in canopy development under water-limited conditions (Supplementary Table S4).

Interestingly, many of these associations, particularly those involving shoot traits, were most prominent at early tillering, suggesting that this stage may be critical for identifying genetic control points. Haplotype b002248 (7H) was one exception, being linked to shoot biomass at later stages, including stem elongation and flowering. Importantly, haploblock b001340 (5H), aligned with the *EGT2* gene, was consistently associated with both RSA and canopy traits across seasons (Supplementary Table S3). This haploblock demonstrated strong pleiotropic effects, being associated with four RSA traits and three canopy traits, including biomass at early tillering, stem elongation, and flowering. These results suggest that this region may regulate shoot–root coordination, with similar roles to *PIN* genes in sorghum that are known to control allometric responses between root and shoot development (Borrell *et al.*, 2022). *EGT2*, located within the b001340 haploblock, plays a critical role in regulating root gravitropism and angle in barley, influencing access to deep soil moisture (Fusi *et al.*, 2022). The detection of this locus in both years and across multiple above- and below-ground traits provides strong field-based evidence of its broader functional role.

In addition to b001340, haploblocks b001081 (4H) and b001856 (6H) were also consistently associated with RSA traits across both seasons, underscoring their stability and potential as robust targets for root-focused breeding strategies under variable environments. Several other haploblocks overlapped with previously reported genes or QTLs. For example, haploblock b002248 (7H), which was associated with canopy biomass traits in our study, co-located with *WSC10040*, a water-soluble carbohydrate QTL previously reported under drought stress (Diab *et al.*, 2004). Haploblock b000756 (3H), detected in 2024, was linked to deeper root traits and co-located with the QTL *RAQ1* which is associated with seminal root angle (Robinson *et al.*, 2016). In addition, *VRN-B1*, a key regulator of flowering time, was located in close proximity to two haploblocks on chromosome 5H: the root-specific b001495 and the shoot-specific b001502 (Supplementary Fig. S2), supporting previous links between flowering regulation and RSA variation (Voss-Fels *et al*., 2018).

The high-throughput screening approach reported in this study can be used for both RSA and canopy-related traits, offering significant advantages for breeding programmes. While traditional root phenotyping methods are labour-intensive, destructive, and limited to a small number of genotypes, UAV-based phenotyping offers a scalable, non-destructive solution. However, it is crucial to understand the value of RSA traits in diverse environmental contexts to effectively include these traits in selection strategies. This remains a somewhat unresolved research question for many crops. Simulation studies have successfully reported enhanced yield in wheat when the crops have an improved ability to access soil water during grain filling (Manschadi *et al.*, 2006; Christopher *et al.*, 2013). The data generated from our study could similarly be used to improve the prediction accuracy of simulation models such as APSIM and evaluate the impact of RSA traits on yield in different scenarios (Hammer *et al.*, 2010). Predicted RSA traits could be integrated into genomic selection models to improve prediction accuracy and enable plant breeders to evaluate RSA contribution to yield and better understand genotype by environment interactions. Furthermore, predicted RSA traits could be integrated into phenomic selection indices alongside canopy traits to assist breeders in developing varieties tailored to specific target environments.

This research provides a novel framework for future field-based root trait prediction. While this study benefited from two contrasting seasons of data, further validation across additional environments and production systems will strengthen the robustness and broader applicability of the approach for other crops. The accuracy of RSA predictions may vary with soil type, seasonal moisture levels, and across different environments and crops, highlighting the need to explore these variables in future studies. Additionally, while this study leveraged multi-spectral UAV imagery and advanced machine learning algorithms, higher resolution imaging (e.g. hyperspectral sensors) and further refinement of model parameters could further enhance predictive accuracy (Smith *et al.*, 2021).

## Conclusion

This study represents a significant step forward in crop phenotyping by integrating UAV-captured VIs with machine learning to evaluate RSA traits for large experimental or breeding panels. Unlike earlier studies that primarily relied on single VIs, such as NDVI or canopy temperature, to indirectly measure or correlate with RSA traits, this approach employed a comprehensive set of VIs obtained through multiple UAV flights, enabling the prediction of root traits at different soil depths. The high accuracy achieved in predicting canopy and RSA traits highlights the potential of this high-throughput, non-destructive methodology for root-based field phenotyping. Moreover, the detection of haploblocks containing functional root genes, including *EGT2* and *DRO1*, showcases the potential for this method to perform robust RSA phenotyping at a population scale required for haplotype mapping or genome-wide association studies. From a crop breeding perspective, the scalability has the potential to facilitate evaluation of large breeding populations to support selection for complex RSA traits. This could accelerate genetic gain for adaptive trait combinations required for climate-resilient varieties.

## Supplementary Material

eraf268_Supplementary_Data

## Data Availability

Data supporting this study will be made available by the corresponding authors upon request and approval from InterGrain Pty. Ltd.

## Supplementary data

The following supplementary data are available at *JXB*  online.

Fig. S1. Root count and dry biomass per core section for two barley genotypes.

Fig. S2. Alignment of significant haploblocks with previously reported QTLs for RSA and canopy traits.

Fig. S3. Root count density of 10 cm interval soil depths for Core20 genotypes in 2022 and 2024.

Fig. S4. Biomass prediction accuracy comparing four modelling approaches using different combinations of vegetation indices as predictor variables.

Fig. S5. Principal component analysis of vegetation indices used in biomass prediction models across two seasons.

Fig. S6. Seasonal temperature, rainfall, and simulated plant-available water in 2022 and 2024.

Table S1. Spectral indices and temperature variables ranked by correlation with dry biomass.

Table S2. Summary of predicted root system architecture traits in 2022 and 2024 seasons.

Table S3. Haploblocks in barley classified by traits, with shoot (green), root (brown), and shared (blue).

Table S4. QTLs and genes in barley aligned with haploblocks, including locations and contexts.
