# Supplementary material for: Phenotyping the hidden half: combining UAV phenotyping and machine learning to predict barley root traits in the field
Source: J Exp Bot. 2025 Jun 28;76(17):5161–78. doi: 10.1093/jxb/eraf268 (PMC12587423; doi:10.1093/jxb/eraf268)
Supplement: eraf268_Supplementary_Data [file eraf268_supplementary_data.zip › jexbot314401-file001.pdf]

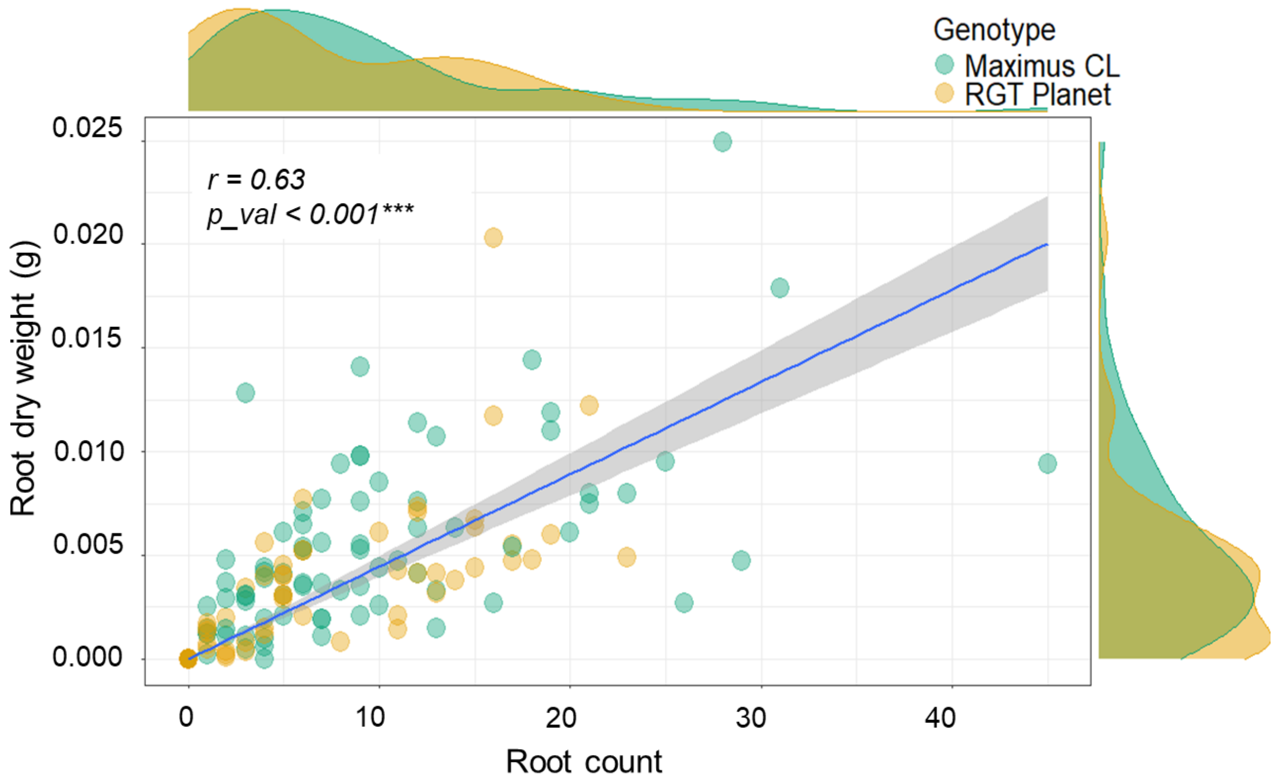

Fig. S1 Positive association between root count and root dry biomass (g) in each section of the soil core for Maximus CL and RGT Planet. Each point represents a soil core section (i.e. at a specific depth), and the colour represents the genotype. The Pearson's correlation (r) value is displayed, with significance levels indicated by (*). Marginal density distributions are presented on the axes to reveal the distribution of root count and root dry biomass (g).

Fig. S2. Alignment of top haploblocks with previously reported QTL for RSA traits and canopy development across the seven chromosomes (1H-7H) of barley. Previously reported QTL on the left of the chromosomes are presented alongside physical positions in Mbp to the right. Shades of green highlights indicate novel significant haploblocks for canopy development (shoot). Shades of brown indicate unique RSA traits of haploblocks. Clear boxes indicate haploblocks from 2022 and hashed (diagonal lines) indicate haploblocks from 2024. Red markers represent key developmental genes.

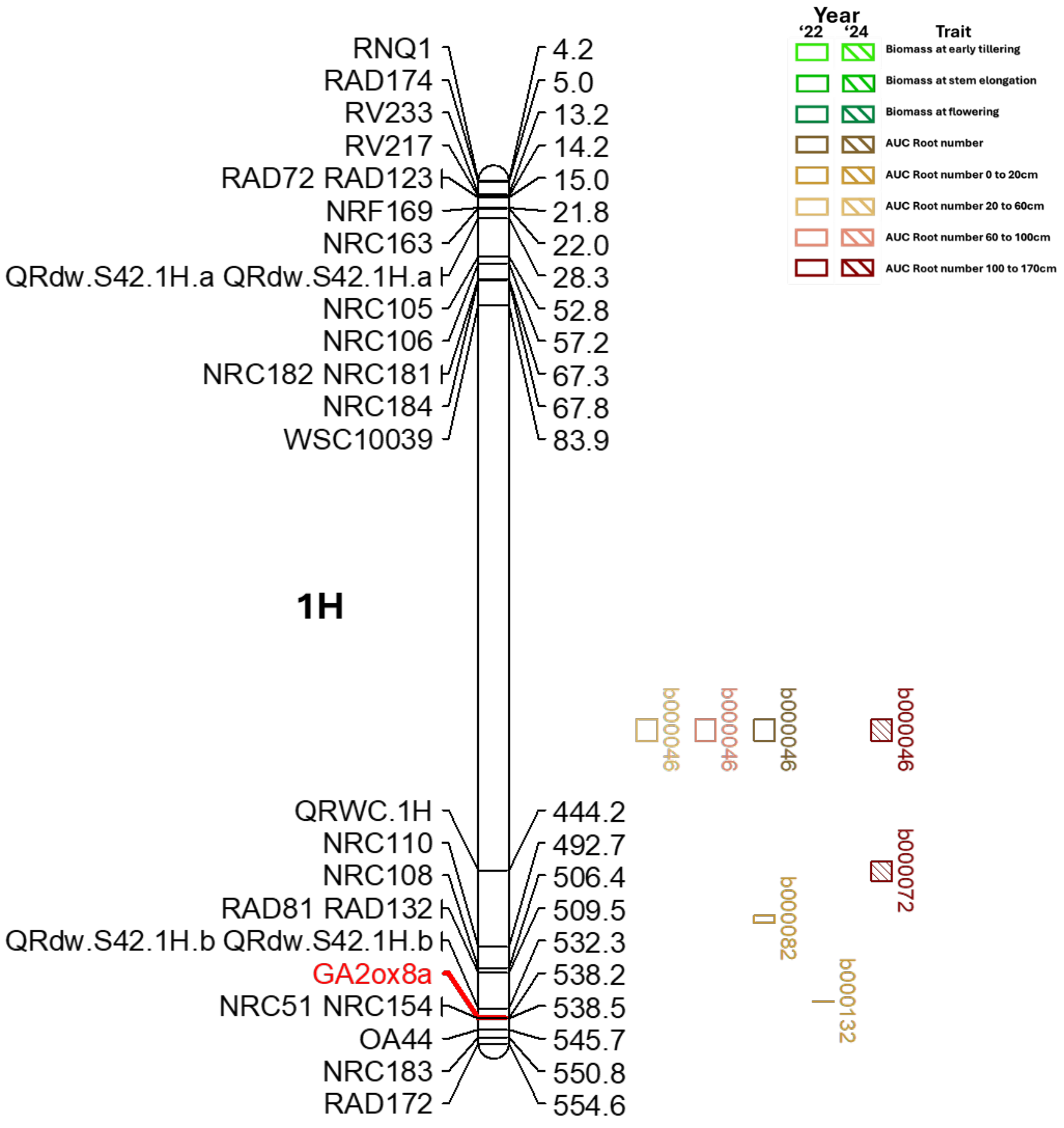

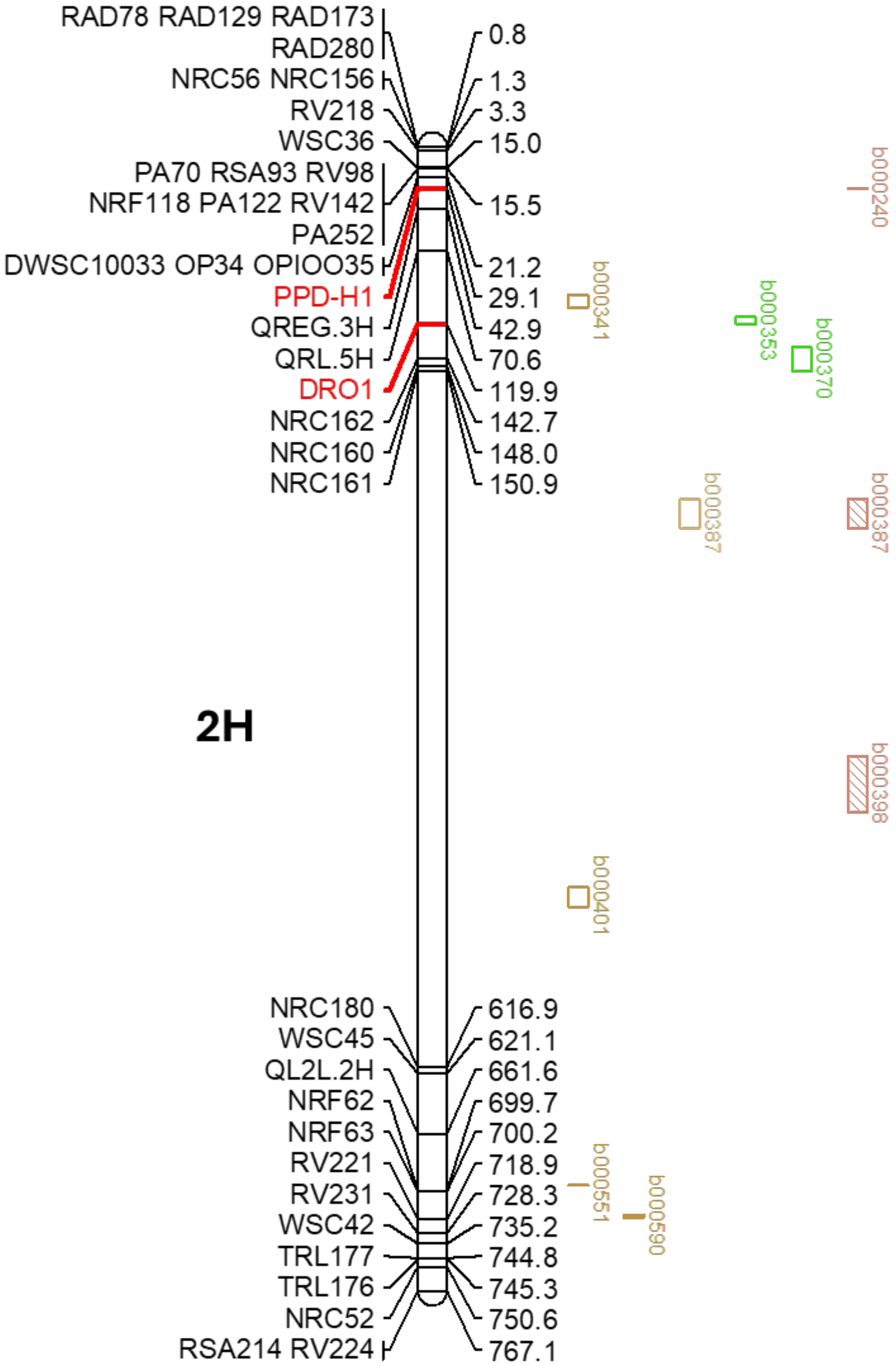
2H
RAD78 RAD129 RAD173
RAD280
NRC56 NRC156
RV218
WSC36
PA70 RSA93 RV98
NRF118 PA122 RV142
PA252
DWSC10033 OP34 OPIOO35
PPD-H1
QREG.3H
QRL.5H
DRO1
NRC162
NRC160
NRC161
NRC180
WSC45
QL2L.2H
NRF62
NRF63
RV221
RV231
WSC42
TRL177
TRL176
NRC52
RSA214 RV224
0.8
1.3
3.3
15.0
15.5
21.2
29.1
42.9
70.6
119.9
142.7
148.0
150.9
616.9
621.1
661.6
699.7
700.2
718.9
728.3
735.2
744.8
745.3
750.6
767.1
b000240
b000341
b000353
b000370
b000387
b000387
b000398
b000401
b000551
b000590

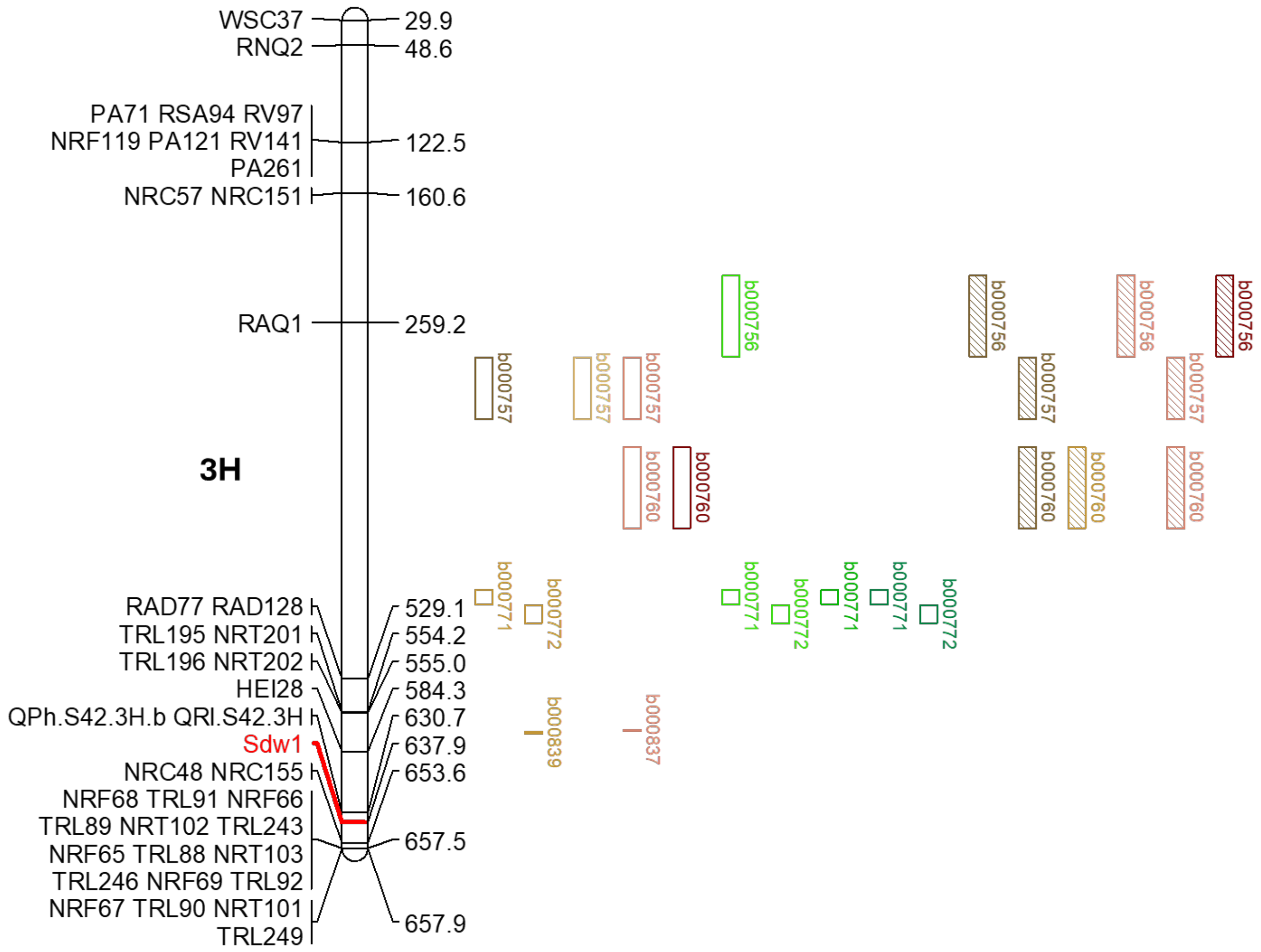
3H
WSC37
RNQ2
PA71 RSA94 RV97
NRF119 PA121 RV141
PA261
NRC57 NRC151
RAQ1
RAD77 RAD128
TRL195 NRT201
TRL196 NRT202
HEI28
QPh.S42.3H.b QRl.S42.3H
Sdw1
NRC48 NRC155
NRF68 TRL91 NRF66
TRL89 NRT102 TRL243
NRF65 TRL88 NRT103
TRL246 NRF69 TRL92
NRF67 TRL90 NRT101
TRL249
29.9
48.6
122.5
160.6
259.2
529.1
554.2
555.0
584.3
630.7
637.9
653.6
657.5
657.9
b000756
b000757
b000760
b000771
b000772
b000839
b000837

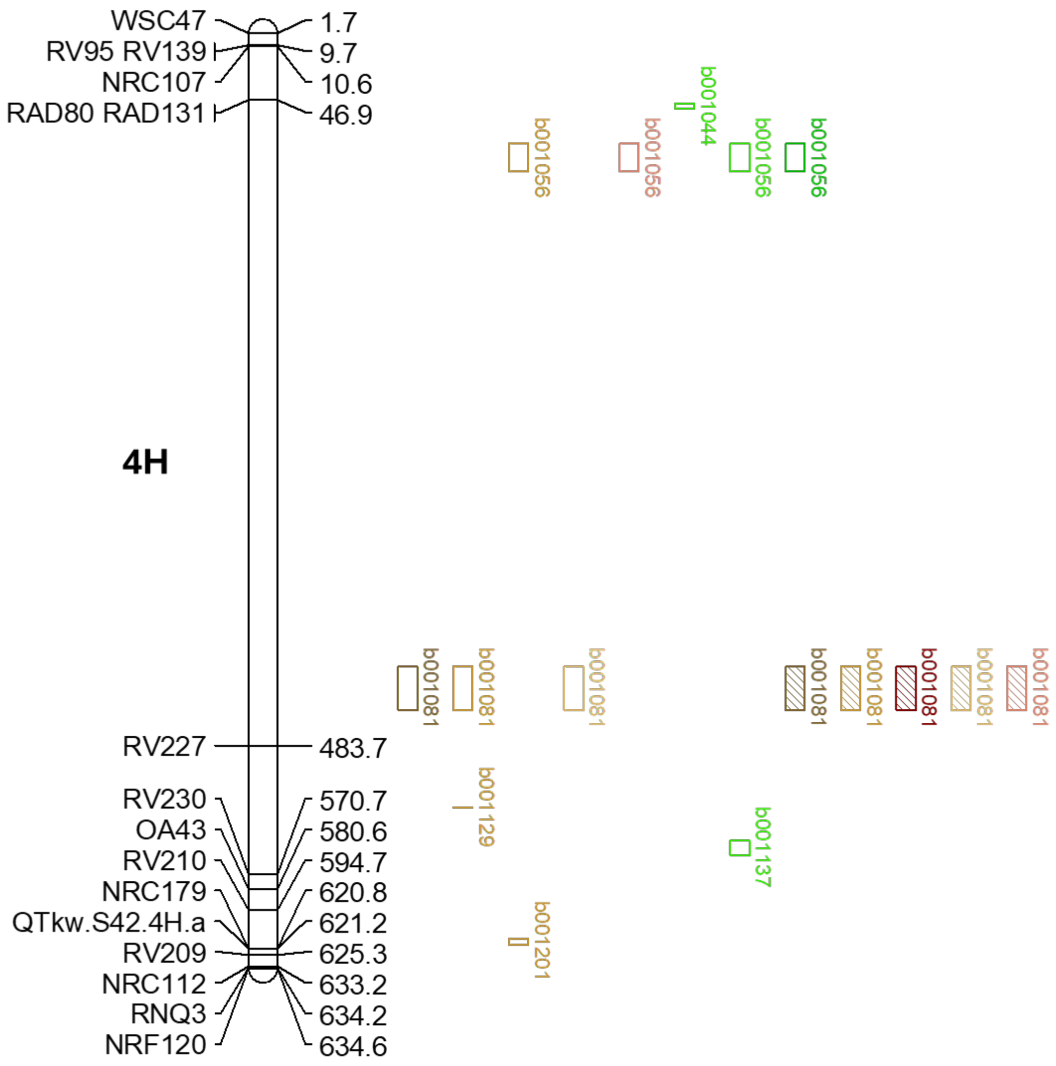
4H
WSC47
RV95 RV139
NRC107
RAD80 RAD131
RV227
RV230
OA43
RV210
NRC179
QTkw.S42.4H.a
RV209
NRC112
RNQ3
NRF120
1.7
9.7
10.6
46.9
483.7
570.7
580.6
594.7
620.8
621.2
625.3
633.2
634.2
634.6
b001044
b001056
b001056
b001056
b001056
b001081
b001081
b001081
b001081
b001081
b001081
b001081
b001081
b001129
b001137
b001201

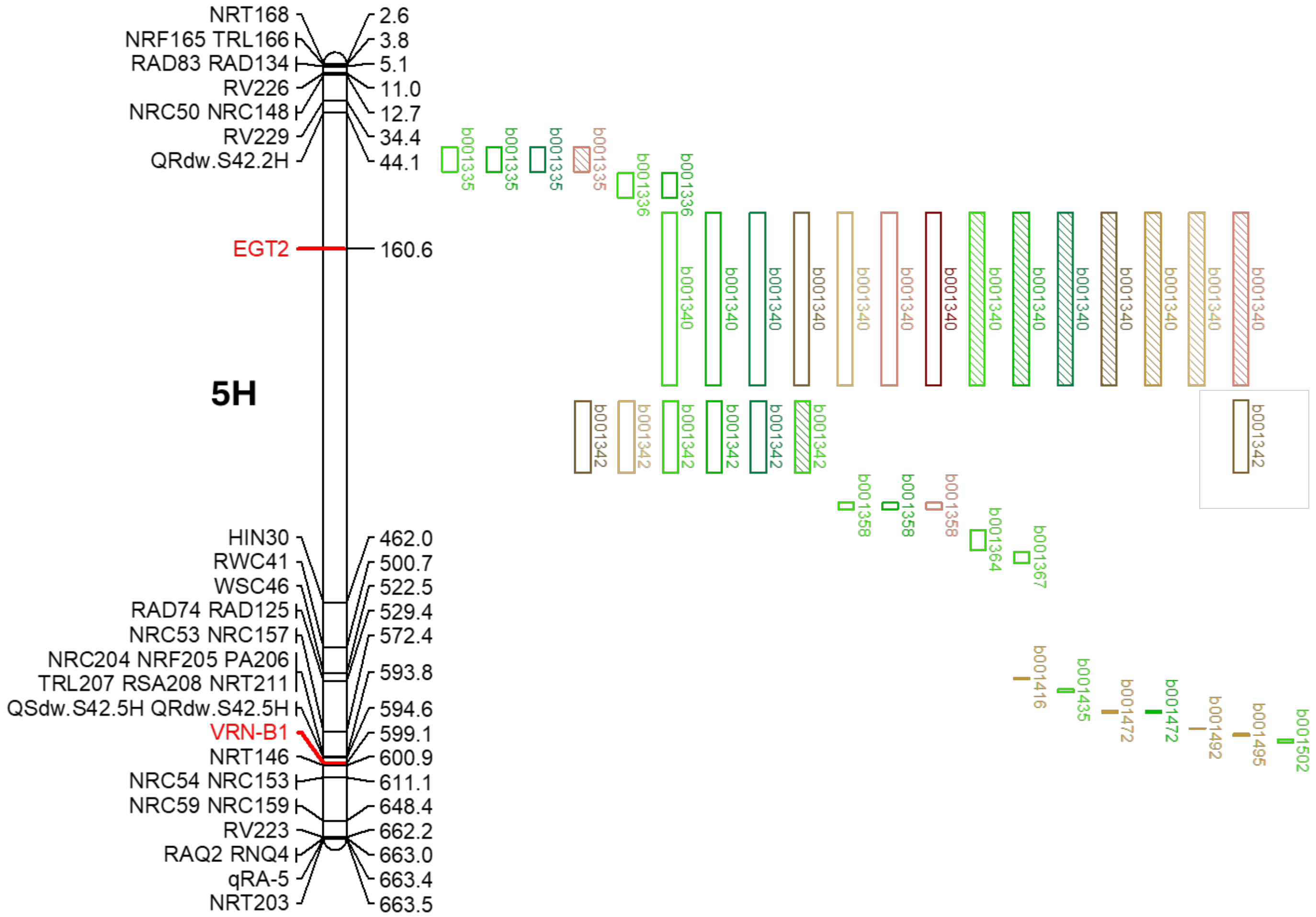
5H
NRT168
NRF165 TRL166
RAD83 RAD134
RV226
NRC50 NRC148
RV229
QRdw.S42.2H
EGT2
HIN30
RWC41
WSC46
RAD74 RAD125
NRC53 NRC157
NRC204 NRF205 PA206
TRL207 RSA208 NRT211
QSdw.S42.5H QRdw.S42.5H
VRN-B1
NRT146
NRC54 NRC153
NRC59 NRC159
RV223
RAQ2 RNQ4
qRA-5
NRT203
2.6
3.8
5.1
11.0
12.7
34.4
44.1
160.6
462.0
500.7
522.5
529.4
572.4
593.8
594.6
599.1
600.9
611.1
648.4
662.2
663.0
663.4
663.5
b001335
b001335
b001335
b001335
b001336
b001336
b001340
b001340
b001340
b001340
b001340
b001340
b001340
b001340
b001340
b001340
b001340
b001340
b001340
b001340
b001342
b001342
b001342
b001342
b001342
b001342
b001342
b001358
b001358
b001358
b001364
b001367
b001416
b001435
b001472
b001472
b001492
b001495
b001502

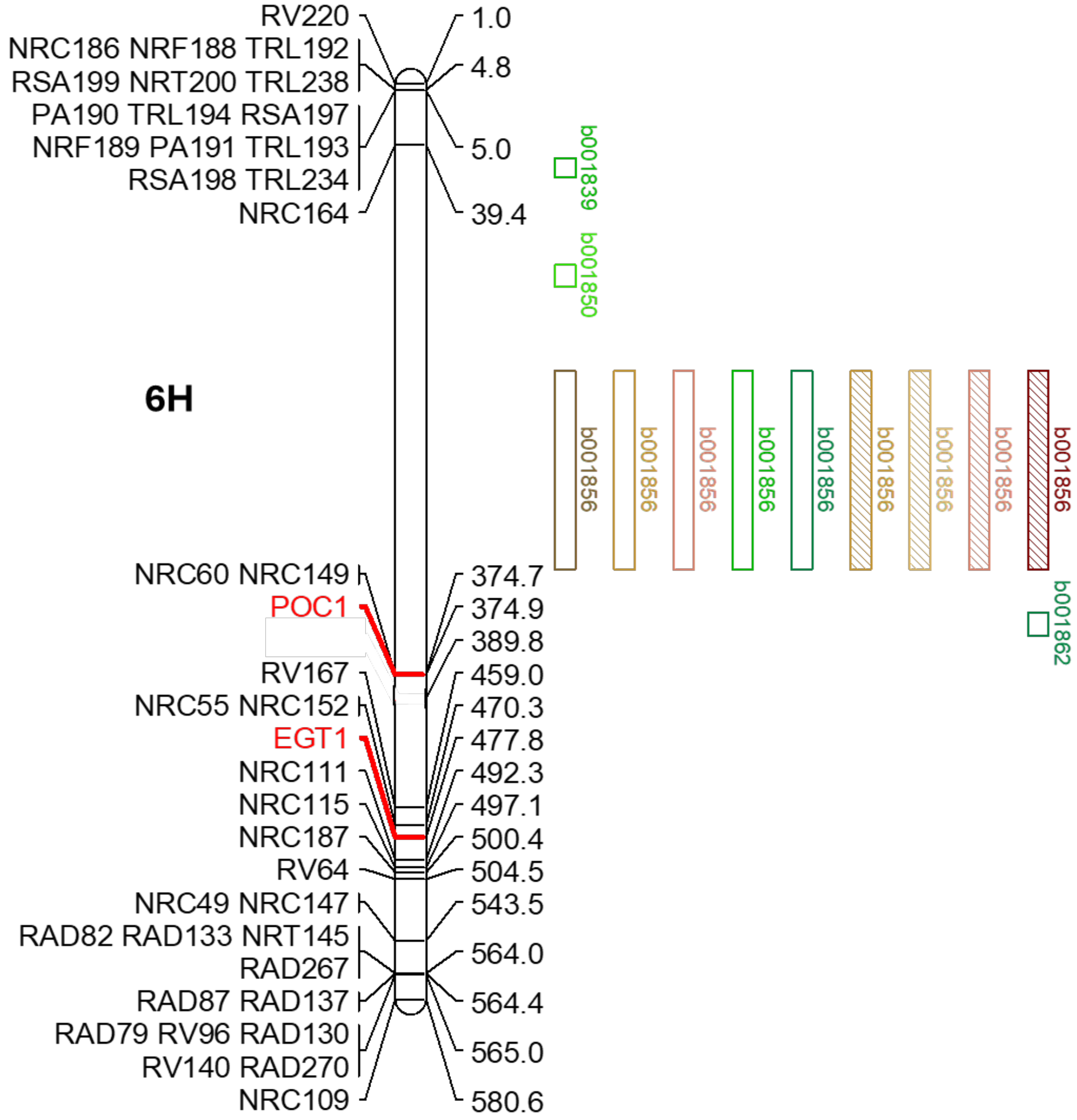
6H
RV220
NRC186 NRF188 TRL192
RSA199 NRT200 TRL238
PA190 TRL194 RSA197
NRF189 PA191 TRL193
RSA198 TRL234
NRC164
NRC60 NRC149
POC1
RV167
NRC55 NRC152
EGT1
NRC111
NRC115
NRC187
RV64
NRC49 NRC147
RAD82 RAD133 NRT145
RAD267
RAD87 RAD137
RAD79 RV96 RAD130
RV140 RAD270
NRC109
1.0
4.8
5.0
39.4
374.7
374.9
389.8
459.0
470.3
477.8
492.3
497.1
500.4
504.5
543.5
564.0
564.4
565.0
580.6
b001839
b001850
b001856
b001856
b001856
b001856
b001856
b001856
b001856
b001856
b001856
b001862

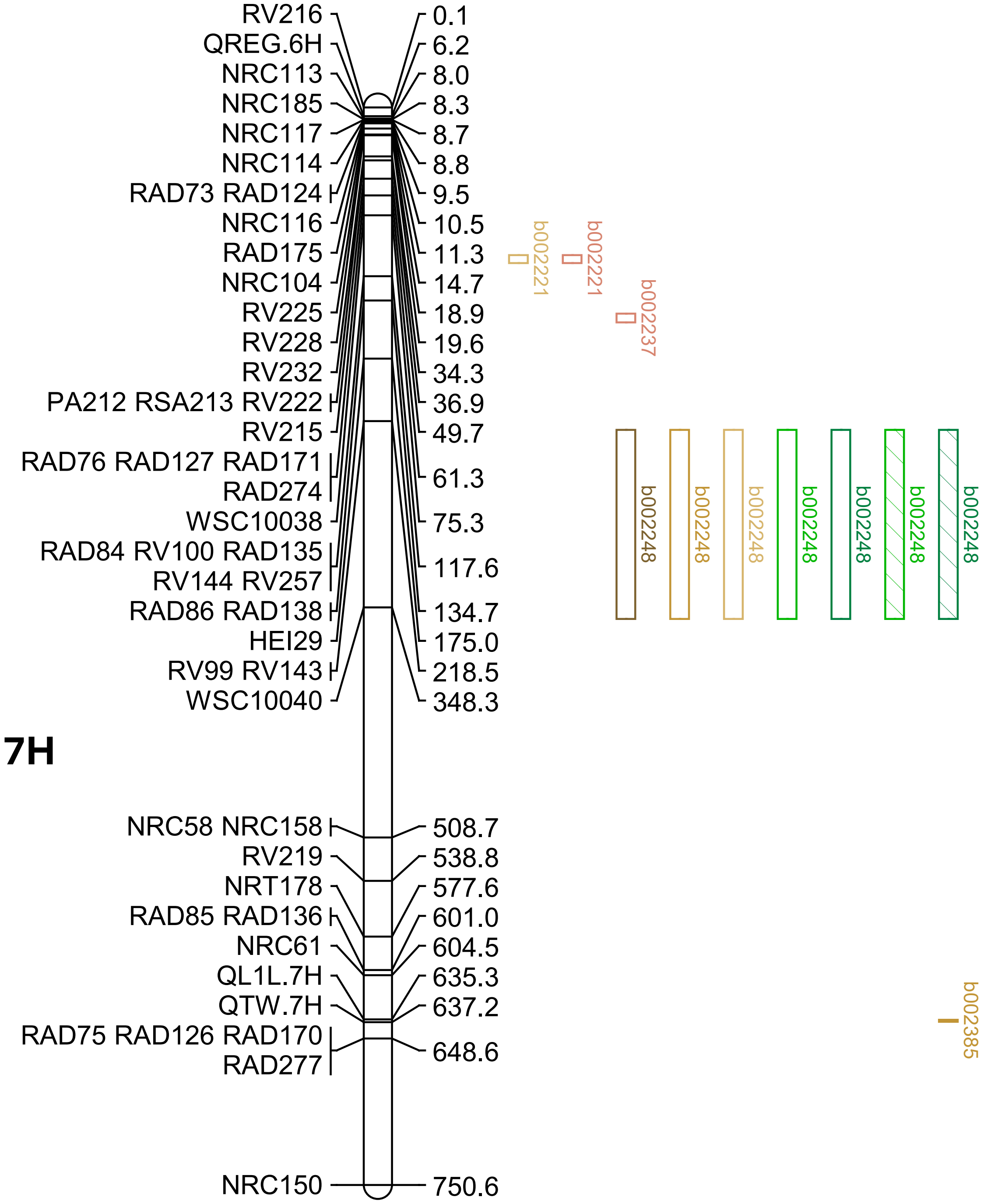

7H
RV216
QREG.6H
NRC113
NRC185
NRC117
NRC114
RAD73 RAD124
NRC116
RAD175
NRC104
RV225
RV228
RV232
PA212 RSA213 RV222
RV215
RAD76 RAD127 RAD171
RAD274
WSC10038
RAD84 RV100 RAD135
RV144 RV257
RAD86 RAD138
HEI29
RV99 RV143
WSC10040
NRC58 NRC158
RV219
NRT178
RAD85 RAD136
NRC61
QL1L.7H
QTW.7H
RAD75 RAD126 RAD170
RAD277
NRC150
0.1
6.2
8.0
8.3
8.7
8.8
9.5
10.5
11.3
14.7
18.9
19.6
34.3
36.9
49.7
61.3
75.3
117.6
134.7
175.0
218.5
348.3
508.7
538.8
577.6
601.0
604.5
635.3
637.2
648.6
750.6
b002221
b002221
b002237
b002248
b002248
b002248
b002248
b002248
b002248
b002248
b002385

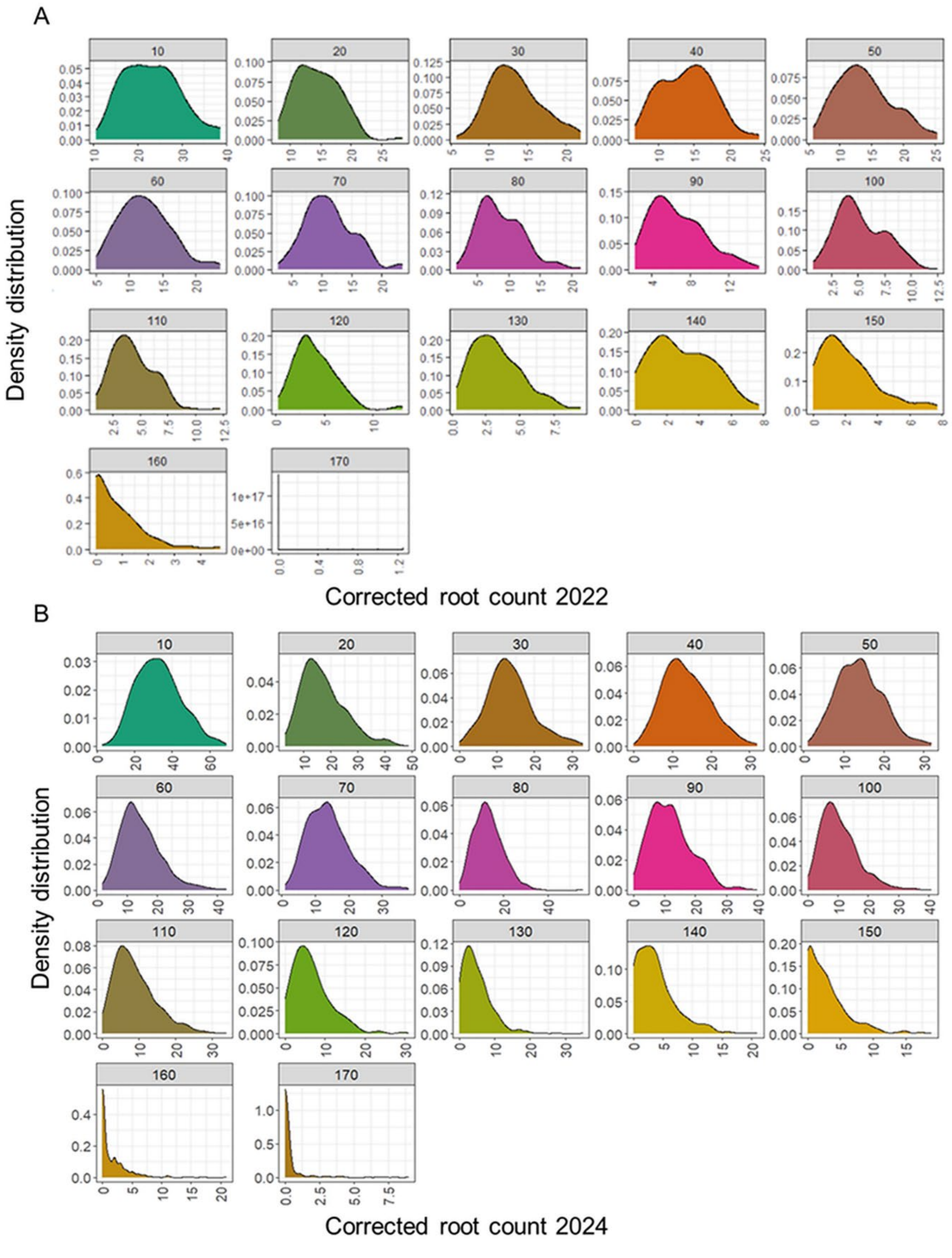

Fig. S3 Root number density distribution across soil depth for 20 genotypes in the Coring Trial. A) 2022 and B) 2024, soil depths from 10 cm to 170 cm and 10 cm intervals. The graph displays adjusted root count density at various soil core sections, showing a decreasing trend in root abundance with depth. Deeper soil layers exhibit a leftward skew in the distribution, reflecting fewer roots at greater depths across both years.

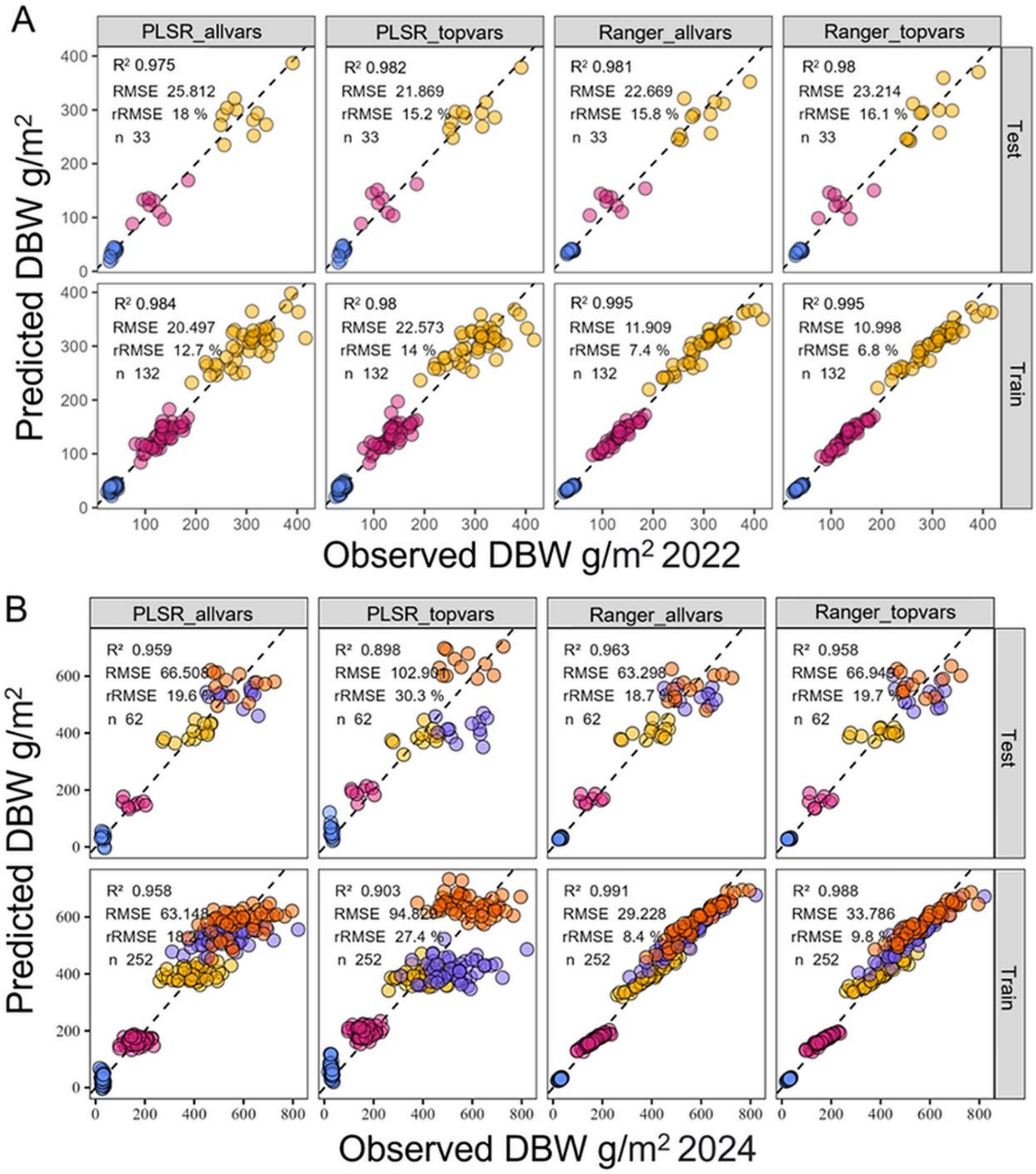

Fig. S4 Predicted above-ground dry biomass using four modelling approaches: Partial Least Squares Regression with all vegetation indices (PLSR_allvar), PLSR using the most important variables (PLSR_topvars), Random Forest with all variables (Ranger_allvars), and Random Forest using the most important variables (Ranger_topvars). (A) Observed versus predicted above-ground dry biomass for 2022 using UAV vegetation indices calculated at three time points. (B) Observed versus predicted above-ground dry biomass for 2024 using UAV vegetation indices calculated at five key developmental stages. The horizontal X-axis represents the manually measured shoot dry biomass (SDB) at ground level, and the vertical Y-axis represents the predicted SDB for the set of 20 genotypes obtained from each model. Model performance is summarised by the correlation coefficient (*r*), residual mean square error (RMSE), relative RMSE (rRMSE), and the number of observations (*n*).

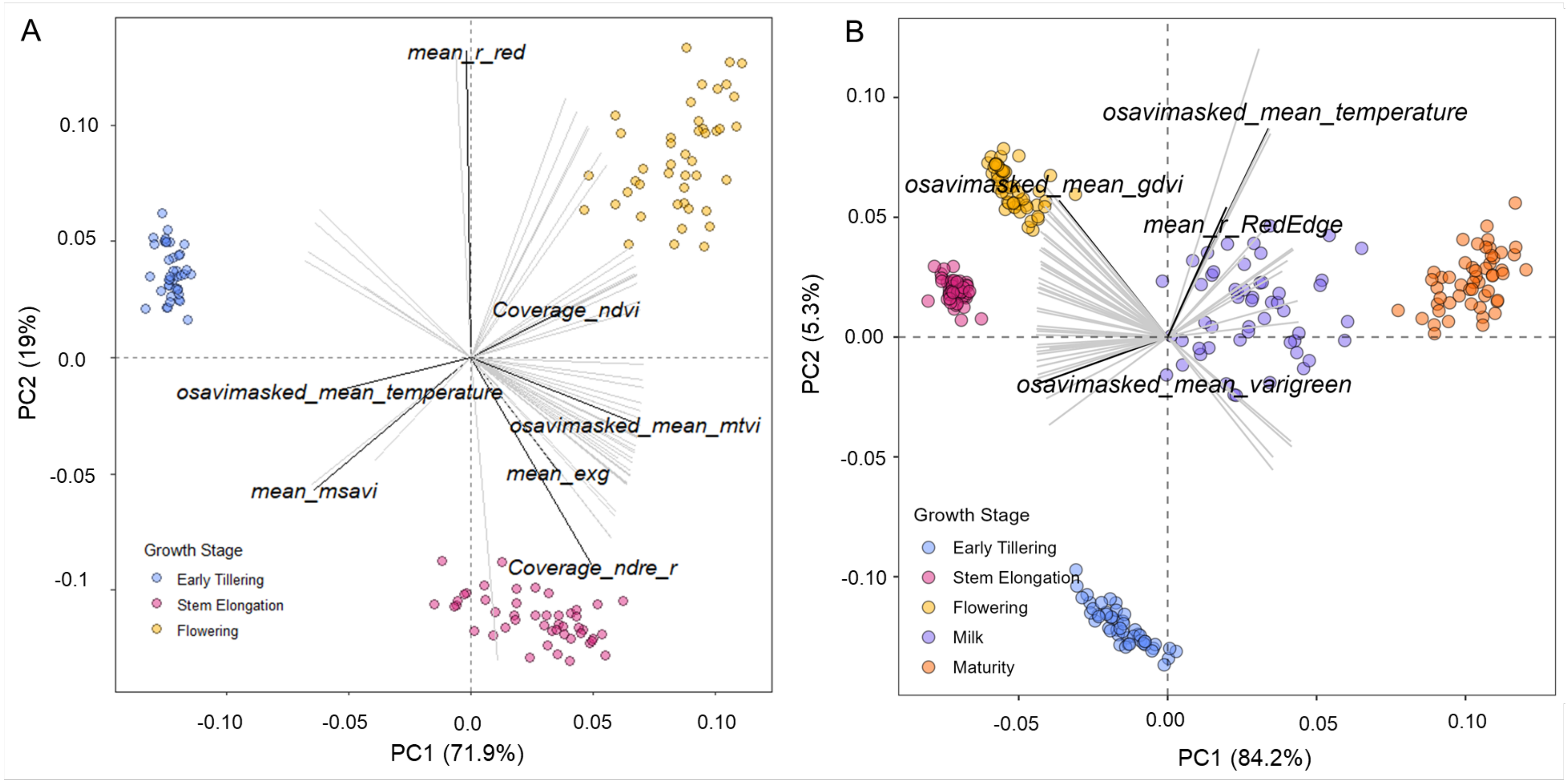

Fig. S5 Biplot of vegetation indices used to predict biomass across multiple growth stages in 2022 and 2024. A) Principal component analysis (PCA) of various vegetation indices used to train PLSR models used for predicting SDB at three distinct growth stages: Early Tillering, Stem Elongation, and Flowering. B) PCA of vegetation indices contributed most to the random forest FR model for predicting SDB at five growth stages in 2024: Early Tillering, Stem Elongation, Flowering, Grain Fill and Maturity.

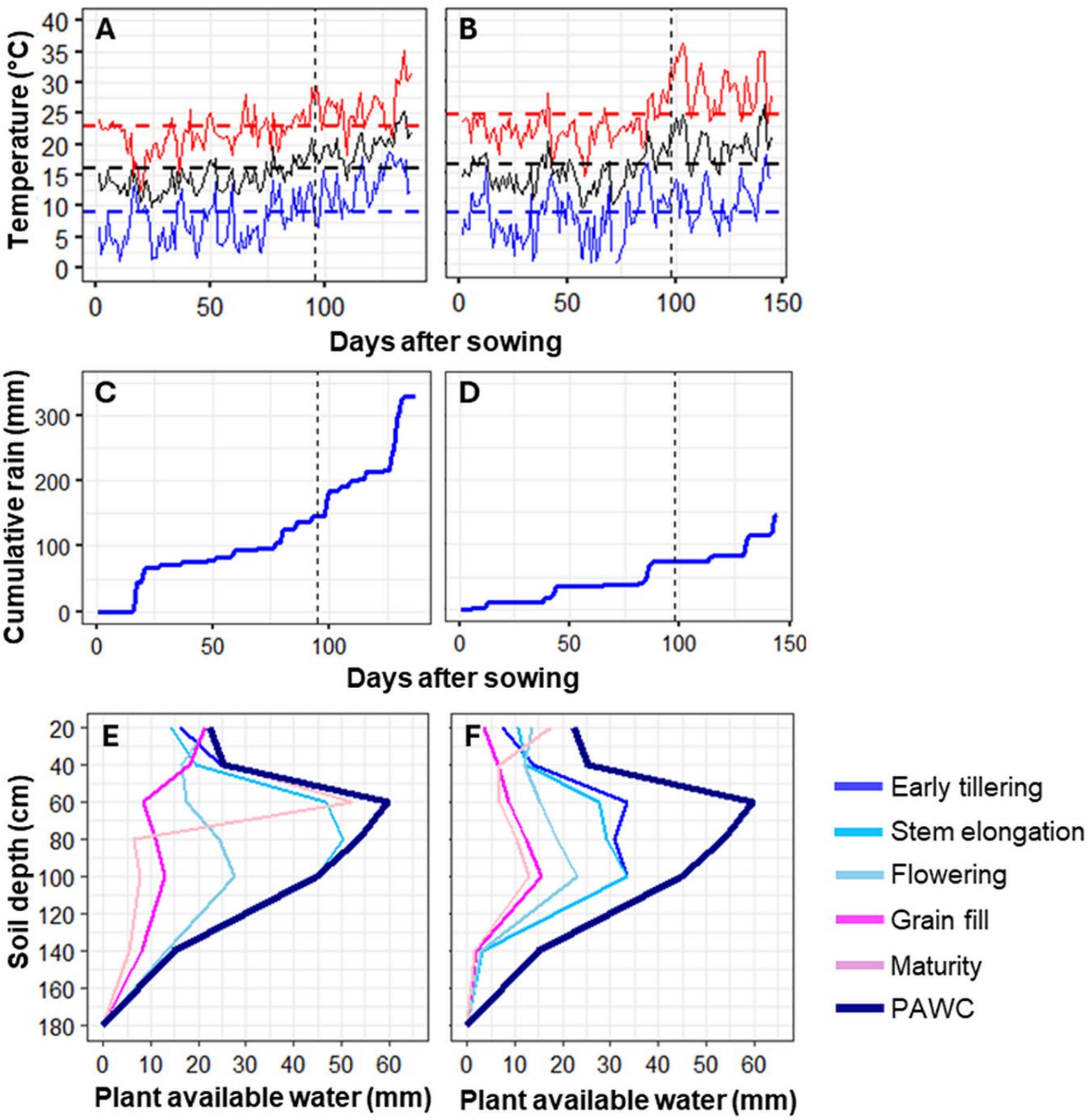

Fig. S6 Daily temperature (A, B) and cumulative rainfall (C, D) over the growing season together with simulated plant available water down along the soil profile at five growth stages (E, F) in 2022 (A, C, E) and 2024 (B, D, F) growing seasons. In (A, B), daily maximum (red), mean (black) and minimum (blue) temperatures are presented together with their respective seasonal averages (horizontal dashed lines). In (E, F), the plant available water capacity (PAWC; black lines) for the soil is presented together with plant available water (PAW; coloured lines) at the time of the drone flights. PAW was simulated with the APSIM-Barley model for cv RGT Planet, and is presented for depth intervals 0–20 cm, 20–40 cm, 40–60 cm, 60–80 cm, 80–100 cm, 100–140 cm, and 140–180 cm. The vertical dashed lines correspond to the flowering date of cv RGT Planet.
